# Manipulation of the Tubulin Code Alters Directional Cell Migration and Ciliogenesis

**DOI:** 10.3389/fcell.2022.901999

**Published:** 2022-07-12

**Authors:** Manuel Müller, Lena Gorek, Natalia Kamm, Ralf Jacob

**Affiliations:** ^1^ Department of Cell Biology and Cell Pathology, Philipps-Universität Marburg, Marburg, Germany; ^2^ DFG Research Training Group, Membrane Plasticity in Tissue Development and Remodelling, GRK 2213, Philipps-Universität Marburg, Marburg, Germany

**Keywords:** Tubulin Tyrosine Ligase (TTL), microtubule tyrosination/detyrosination, Focal Adhesion (FA), directional cell migration, epithelial morphogenesis, primary cilia

## Abstract

Conjunction of epithelial cells into monolayer sheets implies the ability to migrate and to undergo apicobasal polarization. Both processes comprise reorganization of cytoskeletal elements and rearrangements of structural protein interactions. We modulated expression of tubulin tyrosin ligase (TTL), the enzyme that adds tyrosine to the carboxy terminus of detyrosinated α-tubulin, to study the role of tubulin detyrosination/-tyrosination in the orientation of cell motility and in epithelial morphogenesis. Oriented cell migration and the organization of focal adhesions significantly lose directionality with diminishing amounts of microtubules enriched in detyrosinated tubulin. On the other hand, increasing quantities of detyrosinated tubulin results in faster plus end elongation of microtubules in migrating and in polarized epithelial cells. These plus ends are decorated by the plus end binding protein 1 (EB1), which mediates interaction between microtubules enriched in detyrosinated tubulin and the integrin-ILK complex at focal adhesions. EB1 accumulates at the apical cell pole at the base of the primary cilium following apicobasal polarization. Polarized cells almost devoid of detyrosinated tubulin form stunted primary cilia and multiluminal cysts in 3D-matrices. We conclude that the balance between detyrosinated and tyrosinated tubulin alters microtubule dynamics, affects the orientation of focal adhesions and determines the organization of primary cilia on epithelial cells.

## Introduction

Active movement of continuous epithelial cell sheets is a central physiological function during tissue repair and wound healing. This process is influenced by the facilitated rearrangement of the microtubule skeleton in leading edge cells (Garcin and Straube, 2019). Posttranslational modifications (PTM) of αβ-tubulin subunits as well as drug treatments alter the interactome of the cytoskeleton and thus affect cellular motility. Among these modifications that define the tubulin code are detyrosination and re-tyrosination at the carboxy-terminus of α-tubulin, α-tubulin acetylation, polyglutamylation, D2-modifications, palmitoylation and phosphorylation (Nieuwenhuis and Brummelkamp, 2019). Defects in PTMs of microtubules, particularly α-tubulin detyrosination, have been implicated in brain disorders and cancer (Mialhe et al., 2001; Soucek et al., 2006; Moutin et al., 2021; Wattanathamsan and Pongrakhananon, 2021). Recently, angiogenesis-related vasohibins (VASH1/2) were identified as a microtubules detyrosination protein, which catalyze tyrosine removal (Aillaud et al., 2017; Nieuwenhuis et al., 2017). Restoration of L-tyrosine at the carboxy-terminus of α-tubulin is catalyzed by tubulin-tyrosine-ligase (TTL), which associates with the curved conformation of the α- and β-tubulin dimer (Raybin and Flavin, 1975; Prota et al., 2013). Experiments with TTL knockout mice have shown the importance of tubulin tyrosination during brain development (Fukushima et al., 2009). The underlying interplay of posttranslationally modified α-tubulin with molecular machinery in the cytoplasm has not been clarified yet. Neither do we know how these modifications modulate physiological 3D tissue development. Candidate proteins to be involved belong to the family of microtubule plus-end proteins which regulate microtubule dynamics and determine their subcellular architecture. The end binding proteins EB1 and EB3 have a GTP-dependent high affinity to EB-binding sites at growing MT ends and guide microtubules to the cell cortex (Nehlig et al., 2017). Evidence for a link between EB1 and posttranslational microtubule modifications comes from endothelial cells where EB1-knockdown affects microtubule acetylation and-detyrosination (Kim et al., 2013). Recent findings support the idea of α-tubulin tail modification-dependent cytoplasmic linker protein of 170 kDa (CLIP170) and EB1 recruitment to growing plus ends of microtubules (Chen et al., 2021). Microtubules reaching the cell cortex can be captured in close proximity to focal adhesions (Seetharaman and Etienne-Manneville, 2019). This process is divided into three complementary steps. First, microtubules are guided towards focal adhesions by interacting with crosslinking proteins. Next, if they are in close proximity to focal adhesions microtubule plus-ends are captured at the cell cortex. This anchorage is finally stabilized by recruitment of additional polypeptides to form cortical protein complexes in the vicinity of focal adhesions. Apart from focal adhesions, EB1 provides anchoring sites for a variety of protein components, thereby adding further layers of cellular functionality to microtubule ends (Nehlig et al., 2017).

In the current study, we used epithelial cells, in which the α-tubulin tyrosinating enzyme TTL has been knocked out, overexpressed or reconstituted to study the effect of tubulin-detyrosination on cell migration and epithelial morphogenesis. MDCK cells devoid of TTL exhibited an increase in directionality of movement, which was also reflected in the positioning and stability of focal adhesions. Opposing effects were observed in cells that overexpress the α-tubulin modifying enzyme. Furthermore, TTL-knockdown shifts the EB1-interaction pattern from tyr-to detyr-tubulin enriched microtubules, which represents a major posttranslational α-tubulin modification in the absence of TTL. Pulldown experiments revealed interaction of detyr-tubulin with end-binding protein 1 (EB1), integrin-linked kinase (ILK) and β1-integrin. In non-differentiated and in fully differentiated epithelial cells growing in 3D collagen matrices, EB1-GFP positive microtubule tips move faster after TTL-knockout, indicating that elevated detyr-tubulin concentrations facilitate plus end elongation of microtubules. A combination of TTL-knockout and ILK-knockdown decreased cellular motility and focal adhesion stability towards wild type levels, suggesting that the integrin-ILK complex interacts with detyrosinated microtubules through EB1 as mediator. EB1 also accumulates at the base of primary cilia at the apical cell pole of fully polarized MDCK cells. Their subsequent examination revealed correlation between cilia length and detyr-tubulin concentrations. A drastic shortening of primary cilia in cells overexpressing TTL modifies the tubulation pattern of MDCK cells in 3D culture and results in the formation of multiluminal cysts. In conclusion, modulation of TTL concentrations alters the pattern of epithelial cell motility and morphogenesis.

## Materials and Methods

### Antibodies and DNA Constructs

The following monoclonal antibodies were used: anti-α-tubulin (Clone DM 1A; Sigma-Aldrich), anti-acetylated α-tubulin (Clone 6-11B-1; Sigma-Aldrich), anti-EB1 (Santa Cruz), anti-EB1 (Sigma Aldrich), anti-tyrosinated α-tubulin (YL1/2, Santa Cruz) and anti-vinculin (Sigma-Aldrich). The following polyclonal antibodies were used: anti-β-catenin (Sigma-Aldrich), anti-β1-integrin (GTX128839; GeneTex), anti-detyrosinated α-tubulin (Millipore), anti-ILK (4G9, Cell Signaling), anti-MCAK (Thermo Fisher Scientific) and anti-GAPDH (HyTest). Control rabbit and mouse IgG was purchased from Santa Cruz. Cruz. Horseradish peroxidase-conjugated secondary antibodies against mouse and rabbit were obtained from Biorad (mouse and rabbit) and Bethyl (rat). Alexa-labeled secondary antibodies were purchased from Thermo Fisher. The plasmid mCherry-Vinculin-N-21 was a gift from Michael Davidson (Addgene plasmid #55160; RRID; Addgene_55160). The plasmid EB1-GFP (JB131) was a gift from Jennifer Tirnauer and Tim Mitchison (Addgene plasmid #39299; RRID:Addgene_39299). The plasmid mCherry-EB1 was a gift from Torsten Wittmann (Addgene plasmid #80603; RRID; Addgene_80603).

### Cell Culture and 3D Cell Culture

MDCK (Madin-Darby Canine Kidney) type II, MDCK_ΔTTL_-, MDCK_TTL-GFP_, MDCK_ΔTTL+TTL-GFP_, MDCK_EB1-GFP_ and MDCK_ΔTTL+EB1-GFP_ cells were cultivated as described before (Müller et al., 2021). Plasmid transfection of MDCK cells was performed with Lipofectamine 2000 (Invitrogen) according to the manufacturer’s instructions. For MDCK cyst formation, trypsinized cells were resuspended in Matrigel (BD Biosciences). 4,000 cells were resuspended in 30 µl of Matrigel and added to precooled cover slips. Afterwards cysts were grown for 7 days with daily renewal of medium.

### Cell Transfection and Nocodazole Treatment

Plasmid transfection and siRNA knockdown of MDCK cells were performed with Lipofectamine 2000 (transfection/Invitrogen) or Lipofectamine RNAiMAX (siRNA knockdown/Invitrogen) according to the manufacturer’s instructions. For RNA interference, cells were transfected 1 day after seeding with the following siRNA duplexes (Thermo Fisher Scientific): ILK: 5’-GUC GAG UUG AAA GAC CGC UUC GAG-3’, 5’-CUU CGA GUU ACU CUU AGU GAG ACC-3’; MCAK: 5’-GCU CUU CUC UUC UUC CGG GUC UUG-3’, 5’-CUU CCG GGU CUU GAG ACU UUA CUC -3’. Subsequent experiments were performed at day three after transfection. For nocodazole treatment, cells were chilled on ice and incubated with 33 μM nocodazole (Sigma-Aldrich) for 1 h. Corresponding quantities of dimethyl sulphoxide (DMSO) were added to control cells. Afterwards, cells were warmed to 37°C in the continued absence or presence of nocodazole for 1 h. Finally, cells were prepared for cell lysis or fixed for immunostaining.

### Protein Analysis Procedures, Lysate Preparation and Immunoblotting

For preparation of cell lysates the cells were washed with sterile filtered PBS^++^ (PBS supplemented with 1 mM MgCl_2_ and 1 mM CaCl_2_), collected in lysis buffer (150 mM Tris, pH 8; 150 mM NaCl, 150 mM EDTA, 1% Triton X-100, freshly added protease inhibitor cocktail) after the indicated time intervals and incubated at 4°C on a rotating platform for 30 min. Afterwards samples were centrifuged for 15 min at 17,000 g. The protein concentrations in the supernatants were determined by Lowry method and equal protein amounts (20 µg) were separated by SDS-PAGE using the Hoefer-Mini-VE system (Amersham Pharmacia Biotech) and transferred to nitrocellulose membranes. Membranes were blocked in 5% skimmed milk powder in PBS for 1 h and incubated with primary antibodies overnight at 4°C. Detection was performed with horseradish-peroxidase-conjugated secondary antibodies and ECL reagent (Thermo Fischer Scientific) on an Intas gel imager. The results were quantified using LabImage 1D software and ImageJ (see below).

### Immunoprecipitation

MDCK cells were washed with PBS^++^, collected in PHEM lysis buffer (50 mM PIPES, 50 mM HEPES, 1 mM EDTA, 2 mM MgCl_2_, pH 6.9/2 M glycerol/2% Triton X-100/freshly added protease inhibitor cocktail) by mechanical detachment and incubated at 4°C for 30 min on a rotating platform. After centrifugation (17,000 g for 15 min), cleared lysates were precleared and incubated with anti-EB1 antibodies/protein A-agarose beads for 2 h at 4°C. Non-specific IgG/protein A-agarose beads were used as negative control. Finally, beads were rinsed three times with PHEM washing buffer (50 mM PIPES, 50 mM HEPES, 1 mM EDTA, 2 mM MgCl_2_, pH 6.9), once with PBS and boiled in SDS/PAGE loading buffer for Western blot analysis.

### Immunostaining and Immunofluorescence Microscopy

Cells grown on cover slips or 24-well filter inserts were washed with PBS++ twice and fixed with ice-cold Methanol (5 min) or 4% paraformaldehyde (20 min). Afterwards, cells were permeabilized with 0.1% Triton-X-100 for 20 min and blocked in 5% BSA/PBS^++^ for 1 h. Immunostaining was performed with the indicated primary antibodies in blocking reagent for 2 h or overnight. Secondary antibodies labelled with the indicated Alexa Fluor dyes were applied in PBS^++^ for 1 h. Nuclei were stained with Hoechst 33342. Following incubation, cells were washed with PBS^++^ and mounted with ProLongDiamond (Thermo Fisher). Confocal images were acquired on a Leica STELLARIS equipped with a ×93 glycerol planapochromat objective (Leica Microsystems). For live-cell experiments transfected MDCK cells were imaged in a 37°C incubation chamber using a ×63 oil immersion objective on a Leica DMI8 microscope. Immunofluorescence staining of migrating cells was performed by scratching of confluent monolayers on cover slips. The cells were incubated for 0 h or 6 h after scratching, fixed and stained as indicated.

### Live-Cell Imaging

For live-cell imaging, transfected MDCK, MDCK_ΔTTL_, MDCK_EB1-GFP_ or MDCK_ΔTTL+EB1-GFP_ cells were transfected with indicated plasmid constructs or siRNA as indicated. Cells were grown on glass-bottomed dishes (WillCo Dish) or cover slips. All live-cell experiments were performed at 37°C using a Leica DMI8 LED unit and the emission filters 531/32 or 589/40 (Leica Microsystems). Cells were imaged in live-cell imaging solution (Molecular Probes). For EB1 dynamics analysis the cells were transfected with pEB1-GFP or pmCherry-EB1 as described above. To record the dynamics of EB1-GFP/mCherry-EB1, time lapse images were taken every 3 s for an overall time interval of 253 s. EB1 positive areas were detected with the spot detection function of Imaris (Bitplane) and tracked over time. For live-cell migration experiments MDCK_EB1-GFP_ or MDCK_ΔTTL+EB1-GFP_ cells were transfected with pmCherry-vinculin-N-21. Wound healing scratch assays were performed essentially as described (Müller et al., 2021). Recording of the dynamics of mCherry-vinculin assembly/disassembly was performed by image collection every 60 s for an overall time of 3,720 s. Finally, fluorescence intensities and assembly/disassembly dynamics were detected using ImageJ.

### Quantifications and Statistical Analysis

Band densities of Western blots were measured using LabImage 1D and ImageJ software. Band density values were normalized to GAPDH. The MDCK expression level of β-integrin, EB1 and ILK was set to 1. Directionality of cell migration was analyzed and visualized by TrackingTool Pro (V2.1). Fluorescence intensities of mCherry-vinculin were measured from a minimum of 3 time-lapses in three experiments using ImageJ. Mean-square displacement was determined by analyzing single cell migration in wound healing scratch assays (Saxton and Jacobson, 1997). Spatial and temporal measurements for immunofluorescence analysis (distance, area, line scan and volume measurements) were performed by using ImageJ and the ImageJ Tracking Tool. The proximity of detyr-tubulin and vinculin was quantified by immunofluorescence staining measurements with a threshold of proximity set to < 0.3 µm. Dynamics of EB1-accumulations were detected with the spot detection and tracking function of Imaris (Bitplane). 3D rendering was performed by the Vololcity software package (Quorum Technologies).

## Results

### Focal Adhesion Positioning and Orientation of Cell Migration Following TTL-Knockdown or-Overexpression

**FIGURE 1:**
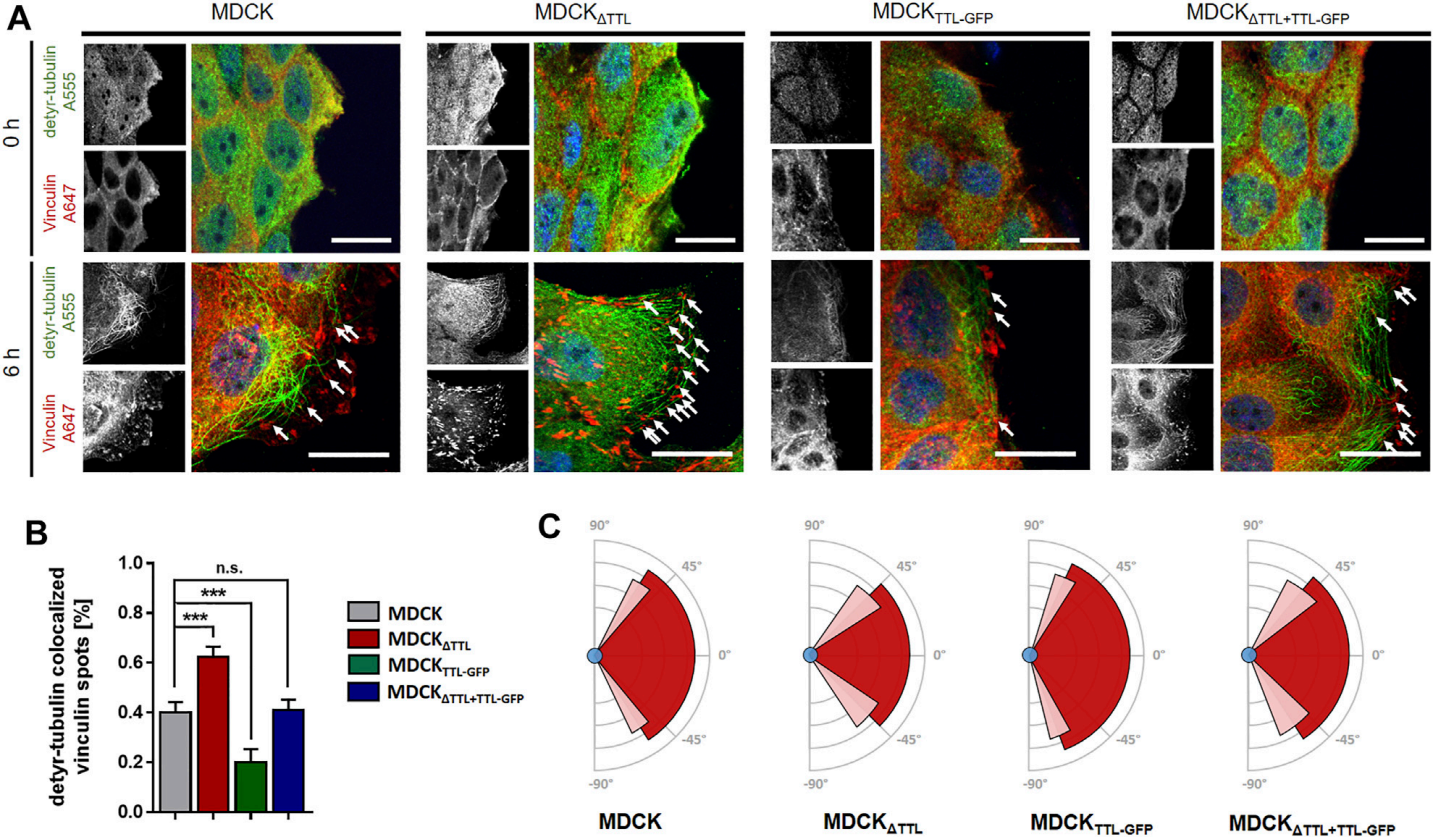
Immunofluorescent staining of detyr-tubulin and vinculin after varying times of monolayer scratching. **(A)** Immunofluorescent staining of detyr-tubulin and vinculin after 0 and 6 h of monolayer scratching. Scratch-induced migration was initiated in confluent monolayers of MDCK, MDCK_ΔTTL,_ MDCK_TTL-GFP_, and MDCK_ΔTTL+TTL-GFP_ 0 and 6 h prior fixation and permeabilization. Cells were stained for focal adhesions using mAb anti-vinculin (Alexa Fluor 647, red) and for visualization of microtubules using pAb anti-detyr-tubulin (Alexa Fluor 555, green). Arrows indicate proximity of detyr-tubulin and vinculin. Scale bars: 20 μm. **(B)** Quantification of detyr-tubulin colocalized vinculin spots after 6 h of migration. Relative amount of colocalized spots at leading edges in MDCK, MDCKΔTTL, MDCKTTL–GFP, and MDCKΔTTL + TTL–GFP cells. Mean ± s.d., *n* = 3 independent experiments per cell line. Statistical significance was tested using one-way ANOVA with Dunnet’s comparison (n.s., not significant; ***p < 0.001). **(C)** Schematic diagram showing the average angle of focal adhesion placement after 6 h of cell migration in MDCK, MDCKΔTTL, MDCKTTL–GFP, and MDCKΔTTL + TTL–GFP cells. Average angles are indicated by dark-red area, SD is depicted in light-red. Angles were measured by ImageJ. Mean ± s.d., *n* = 3 independent experiments per cell line. A total of 10–15 cells were analyzed per experiment.

**FIGURE 2:**
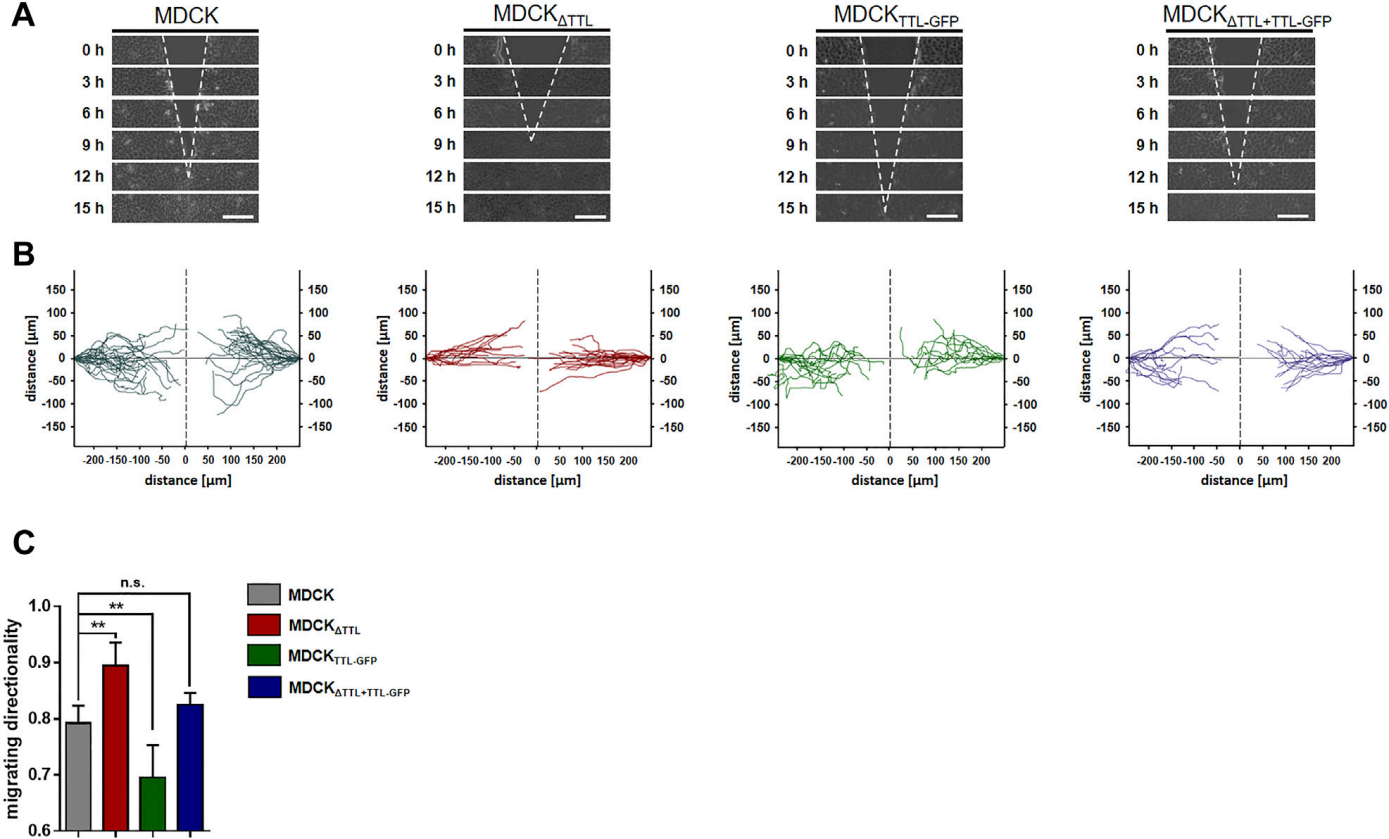
Directionality of cell migration following TTL-modulation. **(A)** Confluent monolayers of MDCK cells were scratch wounded to monitor directional migration. Cells were recorded at 0, 3, 6, 9, 12, and 15 h post-scratching. White dotted lines indicate the wound borders progress over time. Scale bars: 100 µm. **(B)** Single cell migration was recorded and visualized by Tracking Tool™ PRO (Gradientech). One line corresponds to one single cell track **(C)** Cell migration directionality was calculated by Tracking Tool™ PRO. Mean ± s.d., *n* = 4 independent experiments per cell line were performed. Statistical significance was tested using one-way ANOVA with Dunnet’s comparison (n.s., not significant; **p < 0.01).

Mesenchymal cell migration essentially depends on the assembly and disassembly of focal adhesions, which build up physical connections between the extracellular matrix and the actin cytoskeleton through transmembrane receptor integrins (Ridley et al., 2003). The evidence we had shown (Müller et al., 2021) indicates a strong correlation between microtubule detyrosination and focal adhesion assembly. Here, we experimentally manipulated the tubulin detyrosination level in distinct MDCK cell lines to test the causal nature of this relationship. At first, the localisation of focal adhesions was studied by immunofluorescence in MDCK cell lines lacking (MDCK_ΔTTL_) or overexpressing TTL (MDCK_TTL-GFP_) after varying intervals of monolayer scratching (Figure 1A). Especially MDCK_ΔTTL_ cells stretched out their leading edges and projected lamellipodia early after scratching. Already 6 h after scratching these lamellipodia were filled with a network of detyr-tubulin-enriched microtubules that are often spatially linked to vinculin-positive focal adhesions, which was also described for TC-7 kidney epithelial cells (Infante et al., 2000). Moreover, this is consistent with the formation of tubulin-enriched extensions following TTL-knockout in fibroblasts (Peris et al., 2006). MDCK_TTL-GFP_ cells displayed only low signal intensities when stained for detyr-tubulin and rarely exhibited lamellipodia at scratch edges. Quantification of detyr-tubulin-proximal focal adhesions revealed a significant increase in MDCK_ΔTTL_ cells, which was reduced to control levels by ectopic expression of TTL-GFP in MDCK_ΔTTL+TTL-GFP_ cells (Figure 1B). Formation of detyr-tubulin-proximal focal adhesions was lowest in MDCK_TTL-GFP_ cells 6 h after scratching and they were more or less randomly aligned along plasma membranes exposed to the scratch space (Figure 1C, Supplementary Figure S1A,B). On the other hand, in MDCK_ΔTTL_ cells the average angle of focal adhesion placement was much smaller and oriented towards the scratched space indicating efficient focal adhesion assembly in the direction of cell movement to close the cell-free monolayer zone. We then assessed if this directionality in the formation of detyr-tubulin-proximal focal adhesions correlates with directionality of cell movement. TTL-knockout cells closed the scratch faster than MDCK cells expressing TTL [Supplementary Table S1 and (Müller et al., 2021)] and recolonized the scratched region within 9 h after scratching (Figure 2A). At the same time, they migrated with the highest directionality, while TTL-overexpressing MDCK_TTL-GFP_ cells frequently changed their orientation of movement (Figures 2B,C). This is further reflected by mean square displacement (MSD) calculations of cell movement (Supplementary Figure S1C). The slope of the MSD indexes cell motility as a function of time lag (Saxton and Jacobson, 1997). For MDCK_ΔTTL_ cells an initial rise followed by flattening of the curve after 9 h illustrates quick directional movement up to the situation when the scratch is closed and motility is confined. MSD graphs of TTL expressing cells have a minor slope in the initial phase and require more time to reach confinement. This suggests that in the absence of TTL a predominance of microtubules enriched in detyr-tubulin promotes a selective orientation of focal adhesions and directional movement of migrating MDCK cells.

### Interaction of Detyr-Tubulin With EB1 and Focal Adhesion Components

**FIGURE 3:**
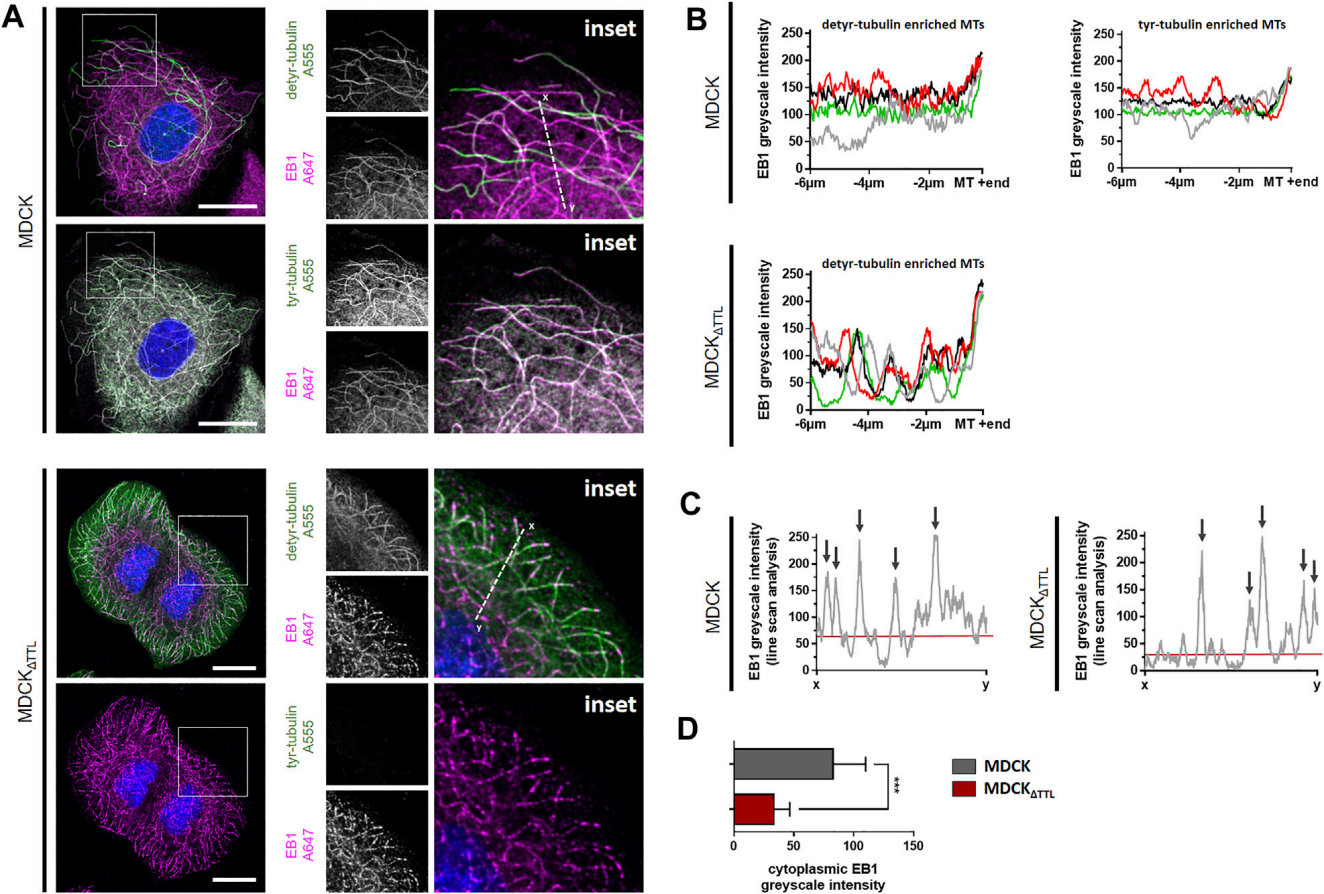
EB1-recruitment along detyr- and tyr-enriched microtubules. **(A)** Subconfluent MDCK and MDCK_ΔTTL_ cells were analyzed by confocal microscopy. Cells were stained for EB1 (Alexa Fluor 647, magenta) and for detyr-tubulin or tyr-tubulin (Alexa Fluor 555, green). Enlarged insets are depicted on the right. Line scans were performed along white dotted lines. Scale bars: 20 µm. **(B)** EB1 intensities were scanned along four detyr- and tyr-enriched microtubules in MDCK cells. For MDCK_ΔTTL_ detyr-enriched microtubules were scanned. Greyscale intensities (0–255) from perinuclear areas to microtubule tips were quantified by ImageJ. Each color indicates the EB1-distibution pattern along one microtubule. **(C)** Line scan analysis of EB1 intensity along white dotted lines as depicted in **(A)** from MDCK and MDCK_ΔTTL_ cells. Greyscale intensities (0–255) were calculated by ImageJ. Arrows indicate line scan crossover with microtubule staining. Red lines show the mean cytoplasmic EB1-intensity. Representative results are depicted. **(D)** Greyscale intensities of EB1 intensities were measured in 20 cytoplasmic regions from MDCK and from MDCK_ΔTTL_ cells. Mean ± s.d., *n* = 3. Statistical significance was tested with Student`s unpaired *t*-test (n.s., not significant; ***p < 0.001).

We next addressed the question if modulations in TTL-expression affect the expression of polypeptides that regulate the dynamics of focal adhesion and microtubules. Antibodies directed against β1-integrin, end binding protein 1 (EB1) and integrin-linked kinase (ILK) were used in immunoblots for quantification of protein levels in our MDCK cell lines lysed in a subconfluent (day 1) or confluent (day 5) condition. Antibodies directed against GAPDH were used as internal reference. No significant alterations could be observed for β1-integrin- or ILK-expression following TTL-knockout or-overexpression. On the other hand, expression of EB1 was significantly enhanced in MDCK_ΔTTL_, MDCK_TTL-GFP_ and MDCK_ΔTTL+TTL-GFP_ cells either early after plating or when growing in a cell monolayer (Supplementary Figure S2A,B). Elevation of EB1-expression following TTL-knockout and-overexpression is astonishing, since both conditions lead to obviously opposing characteristics of cell migration. However, microtubule disruption by nocodazole treatment did not alter the EB1-expression pattern (Supplementary Figure S3), which indicates that the observed alterations in EB1-expression do not depend on the formation or stability of microtubules. If we then analyzed the subcellular distribution of EB1 by immunofluorescence in MDCK and MDCK_ΔTTL_ cells, the protein could be detected on the ends as well as aligned along the lattice of microtubules enriched in detyr- and tyr-tubulin (Figure 3A), which is in agreement with earlier observations (Sandblad et al., 2006; Bieling et al., 2008; Manna et al., 2008). Especially in MDCK cells overlap between EB1- and tyr-tubulin-staining is remarkably high, while EB1- and detyr-tubulin-staining occasionally overlap in this cell line. The pattern obviously changes in MDCK_ΔTTL_ cells, which show sparse tyr-tubulin staining. Here, significant quantities of EB1 concentrate at microtubule ends and punctate clusters distributed along detyr-tubulin enriched microtubules. Scans of EB1-fluorescence intensity along microtubules support the impression of evenly dispersed EB1 molecules on the microtubule lattice in MDCK cells versus discontinuous spreading of prominent EB1 clusters along detyr-tubulin enriched microtubules in MDCK_ΔTTL_ cells (Figure 3B). Moreover, line scan analysis reveals significant cytosolic EB1-staining in MDCK cells, which is less pronounced in MDCK_ΔTTL_ cells (Figures 3C,D). This indicates that TTL-knockdown and the associated shift from tyr-to detyr-tubulin enriched microtubules also shifts the EB1-interaction pattern to these posttranslationally modified microtubules.

**FIGURE 4:**
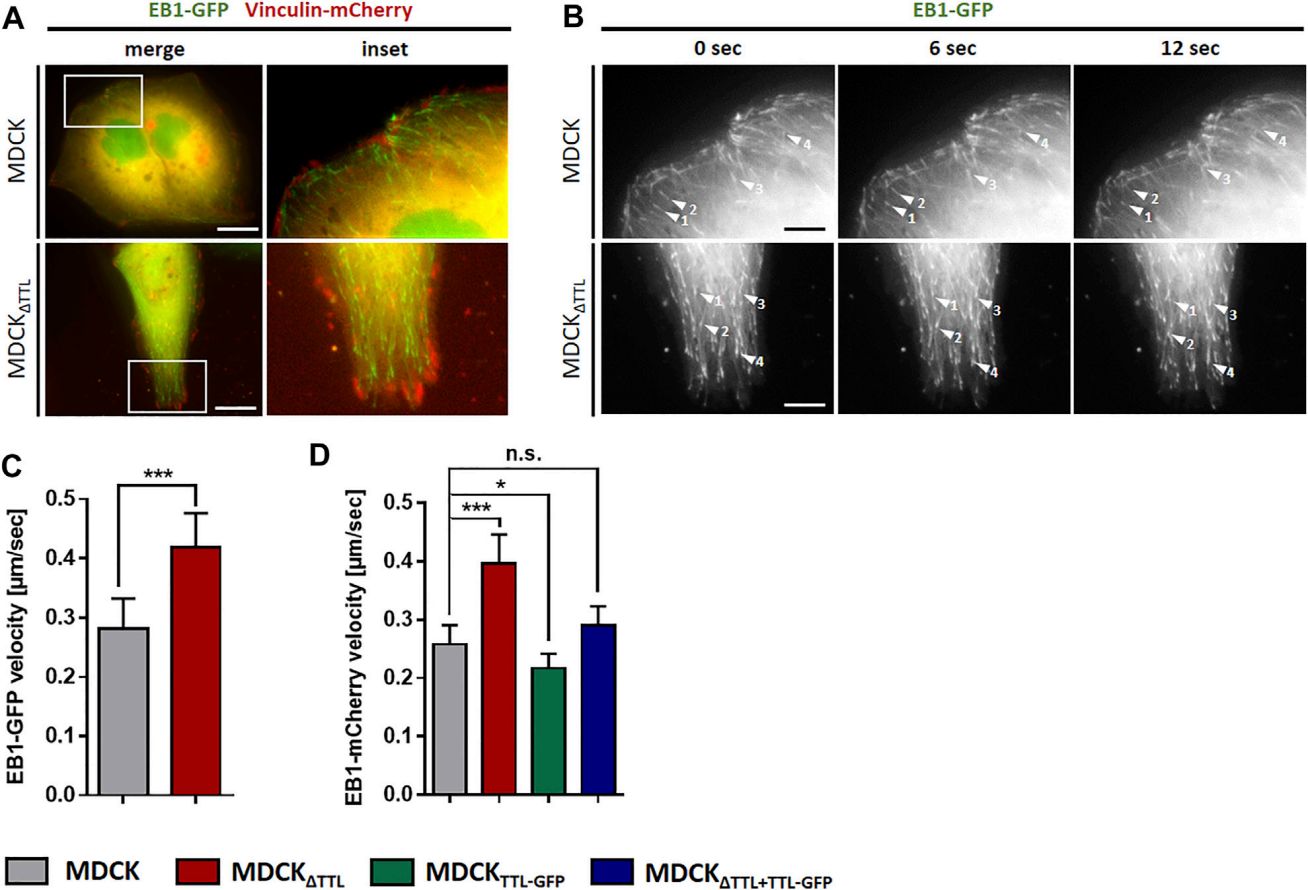
Dynamics of EB1 in MDCK cells. **(A)** Subconfluent living MDCK and MDCK_ΔTTL_ cells transiently transfected with EB1-GFP/vinculin-mCherry incubated on glass bottom WillCo dishes under a humidified atmosphere of 5% CO_2_ in air. Dynamics of EB1-GFP (green) was analyzed by fluorescence microscopy at 37°C. Vinculin-mCherry (red) indicates focal adhesions. Scale bars: 10 µm. **(B)** EB1-GFP dynamics was observed in MDCK and MDCK_ΔTTL_ cells. Time-lapse imaging was recorded over 12 sec. Each arrowhead indicates EB1-GFP comet-like accumulation, tracked for indicated time intervals. Scale bars 2 µm. **(C)** Quantification of EB1-GFP velocities in MDCK and MDCK_ΔTTL_ cells. Experiments were quantified with the Imaris Cell Imaging Software. Mean ± s.d., *n* = 3. Statistical significance was tested with Student`s unpaired *t*-test (***p < 0.001). **(D)** Quantification of EB1-mCherry velocites in MDCK, MDCK_ΔTTL,_ MDCK_TTL-GFP_ and MDCK_ΔTTL+TTL-GFP_ cells, transfected with EB1-mCherry. Mean ± s.d., *n* = 3. Statistical significance was tested with Student`s unpaired *t*-test (n.s., not significant; *p < 0.05; ***p < 0.001).

To determine if this transition alters the formation of plus-tips and EB1-dynamics, the mobility of EB1-GFP-positive comet-like plus-tips was studied in MDCK and MDCK_ΔTTL_ cells co-transfected with EB1-GFP and vinculin-mCherry. Fluorescence microscopic images in Figure 4A show that vinculin-mCherry positive focal adhesion plaques mainly accumulate in the periphery of subconfluent co-transfected cells. EB1-positive microtubule plus ends were heading towards these plaques in both cell lines (Figures 4A,B and Supplementary Movies S10, S11). Investigation of the EB1 plus end movement towards focal adhesions in living cells revealed that the EB1-positive spots moved with a speed of 0.2–0.3 μm/s in MDCK cells. This speed was significantly elevated in MDCK_ΔTTL_ cells to > 0.4 µm/s, which reflects enhanced microtubule growth rates (Figure 4C). This observation could be confirmed if the construct EB1-mCherry was transfected (Figure 4D). Overexpression of TTL-GFP or TTL-reconstitution in MDCK_ΔTTL_ cells decreased the speed of EB1-mCherry positive plus-tips to control levels, indicating that microtubules are stabilized if TTL is knocked out. This could be based on suppression of the depolymerizing motor mitotic centromere-associated kinesin (MCAK) by detyr-enriched microtubules (Peris et al., 2009). In this case, depletion of MCAK would have similar effects on the migration pattern as TTL-knockout. We challenged this scenario by MCAK-knockdown in MDCK and MDCK_ΔTTL_ cells. Subsequently, their migration was recorded in scratch assays (Supplementary Figure S4). Wound closure speed and orientation of movement were reduced in both cell lines if MCAK was depleted, which excludes a major role of MCAK suppression by detyr-tubulin enriched microtubules in the migration patterns following TTL-knockdown. However, it confirms a previously published model of MCAK facilitating robust directional migration as regulator of focal adhesion stability (Zong et al., 2021). We thus asked if the observed efficient microtubule polymerization in MDCK_ΔTTL_ cells affects the stability of focal adhesion plaques? This was addressed by monitoring the assembly or disassembly of mCherry-vinculin positive focal adhesions in migrating MDCK or MDCK_ΔTTL_ cells. Supplementary Figure S5 shows that the dwell time of mCherry-vinculin at focal adhesions of the leading edge of moving cells was considerably higher in MDCK_ΔTTL_ than in MDCK cells, which is in agreement with previously published photoconversion experiments of photoactivatable vinculin-mEOS2 (Müller et al., 2021). Thus, elevated plus end elongation of focal adhesion targeting microtubules is concomitant with increased time intervals of vinculin-residence at focal adhesions indicating improved focal adhesion stability at the cell front if TTL is knocked out.

**FIGURE 5:**
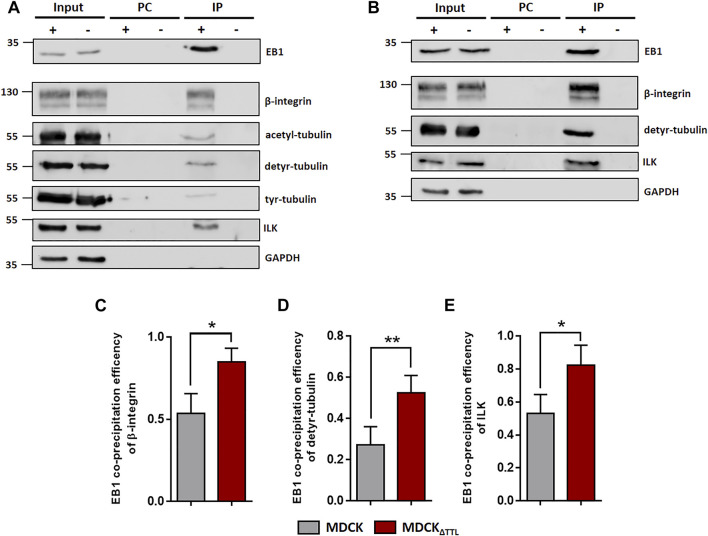
EB1-pulldown following TTL-modulation. **(A)** MDCK cell lysates were incubated with anti-EB1 antibodies followed by precipitation with agarose beads. Precipitates were analyzed by immunoblot using antibodies directed against β1-integrin (CD29), acetyl-, detyr- and tyr-tubulin, ILK, GAPDH and EB1. *n* = 3 independent experiments. PC, Pre-clearing using agarose beads; IP+, Immunoprecipitation using EB1 antibodies and agarose beads; IP-, Immunoprecipitation using unspecific IgG antibodies and agarose beads. Representative results. **(B)** MDCK_ΔTTL_ cell lysates were incubated with anti-EB1 antibodies followed by precipitation with agarose beads. Precipitates were analyzed by immunoblot using antibodies directed against β1-integrin, detyr-tubulin, ILK, GAPDH and EB1. *n* = 3 independent experiments. PC, Pre-clearing using agarose beads; IP+, Immunoprecipitation using EB1 antibodies and agarose beads; IP-, Immunoprecipitation using unspecific IgG antibodies and agarose beads. Representative results. **(C–E)** Quantification of MDCK and MDCK_ΔTTL_ co-precipitation efficiencies of β1-integrin **(C)**, detyr-tubulin **(D)** and ILK **(E)** from three independent experiments. The efficiencies were normalized by the total quantities of each polypeptide in the input. Mean ± s.d., *n* = 3. Statistical significance was tested with Student`s unpaired *t*-test (*p < 0.05; **p < 0.01).

EB1, which interacts with acetylated-, detyr- and tyr-tubulin as assessed by co-immunoprecipitation (Figure 5), also pulls down a central regulatory element for focal adhesion stability, the ILK complex, which is consistent with data from Akhtar and Streuli (Akhtar and Streuli, 2013). This co-precipitation was significantly more efficient if TTL was knocked out from MDCK cells and accordingly depleted if TTL was overexpressed (Figures 5B–E; Supplementary Figure S6). We thus concentrated on the role of the integrin-ILK complex in EB1-recruitment to focal adhesions. It has been shown that in the absence of ILK, EB1 does not form complexes with β1-integrin (Akhtar and Streuli, 2013). In MDCK and MDCK_ΔTTL_ cells total quantities of EB1 or β1-integrin were not affected by RNAi-dependent specific depletion of ILK, which also points to a post translational regulation mediated by ILK (Supplementary Figure S7A,B). Interestingly, increased quantities of focal adhesions following TTL-knockout (Müller et al., 2021) were significantly reduced by ILK-silencing in MDCK cells (Supplementary Figure S7, S8). Moreover, ILK-depletion decreased elevation of focal adhesion stability in MDCK_ΔTTL_ cells (Supplementary Figure S7D), suggesting that ILK plays a central role in alterations of focal adhesion organization observed by TTL-knockout. This conclusion was confirmed by ILK-knockdown-mediated reduction in the migration velocity of MDCK_ΔTTL_ cells (Supplementary Figure S7E,F). We thus claim a scenario in which EB1 recruits enhanced quantities of detyr-tubulin enriched microtubules in MDCK_ΔTTL_ cells to the integrin-ILK complex for fast and directed migration of non-polarized MDCK cells.

### Ciliogenesis and Epithelial Morphogenesis in 3D Culture

**FIGURE 6:**
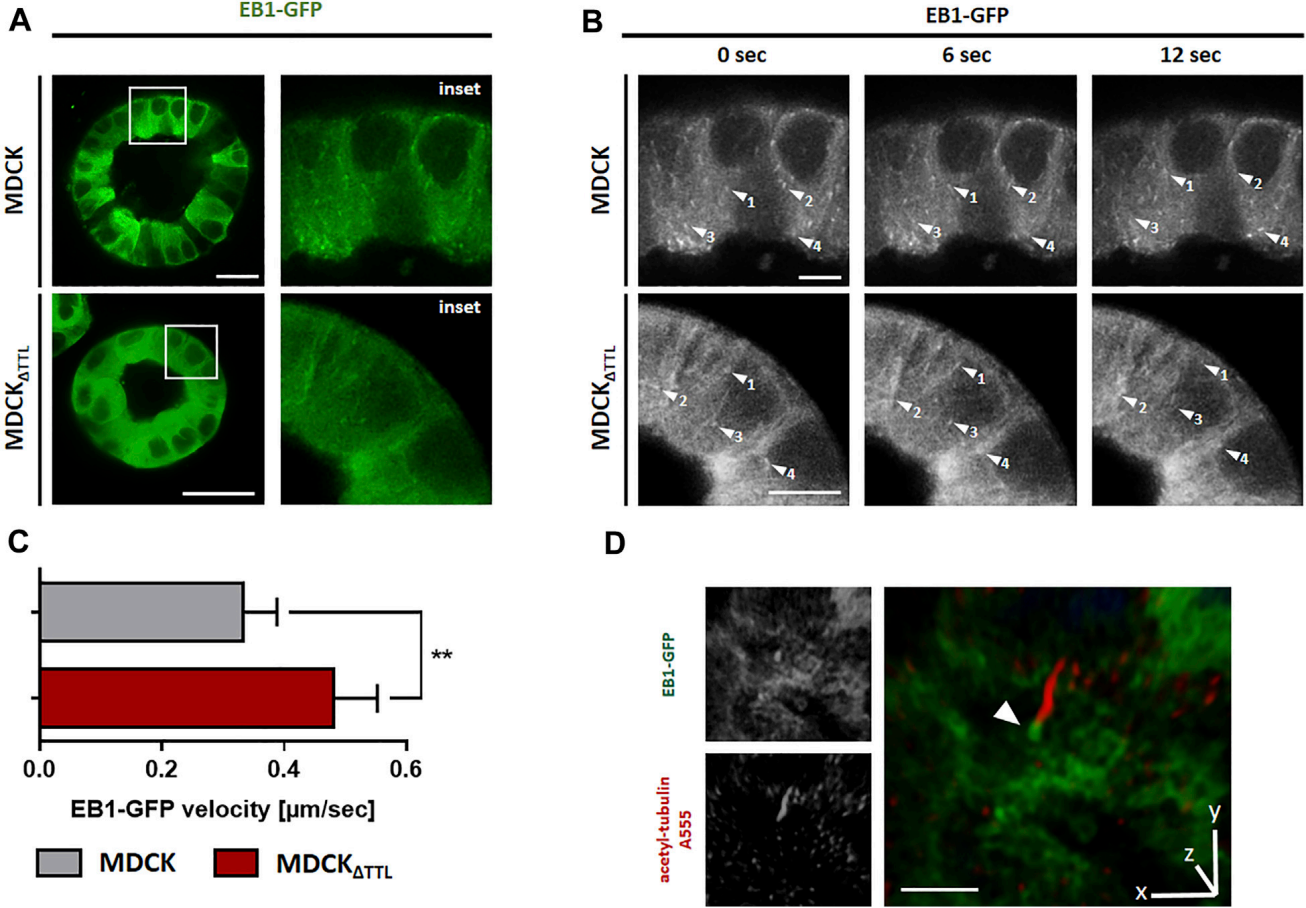
TTL-knockdown promotes EB1 dynamics in MDCK cysts. **(A)** Living MDCK and MDCK_ΔTTL_ cells stably transfected with EB1-GFP were cultured in Matrigel for 7 days. Dynamic of EB1-GFP (green) was analyzed by fluorescence microscopy at 37°C and a humidified atmosphere of 5% CO_2_. Scale bars: 20 µm. **(B)** Time-lapse imaging was recorded for 12 s. Each arrowhead indicates EB1-GFP comet-like accumulation, traced for several time points. Scale bars 4 µm. **(C)** Quantification of EB1-GFP velocities in MDCK and MDCK_ΔTTL_ cysts. Experiments were quantified with the Imaris Cell Imaging Software. Mean ± s.d., *n* = 3. Statistical significance was tested with Student`s unpaired *t*-test (**p < 0.01). **(D)** 3D-rendered image of acetylated tubulin staining (primary cilium, red) in EB1-GFP (green) expressing MDCK_ΔTTL_ cyst. Arrowhead indicates accumulation of EB1 around the ciliary base. Scale bar: 5 µm.

**FIGURE 7:**
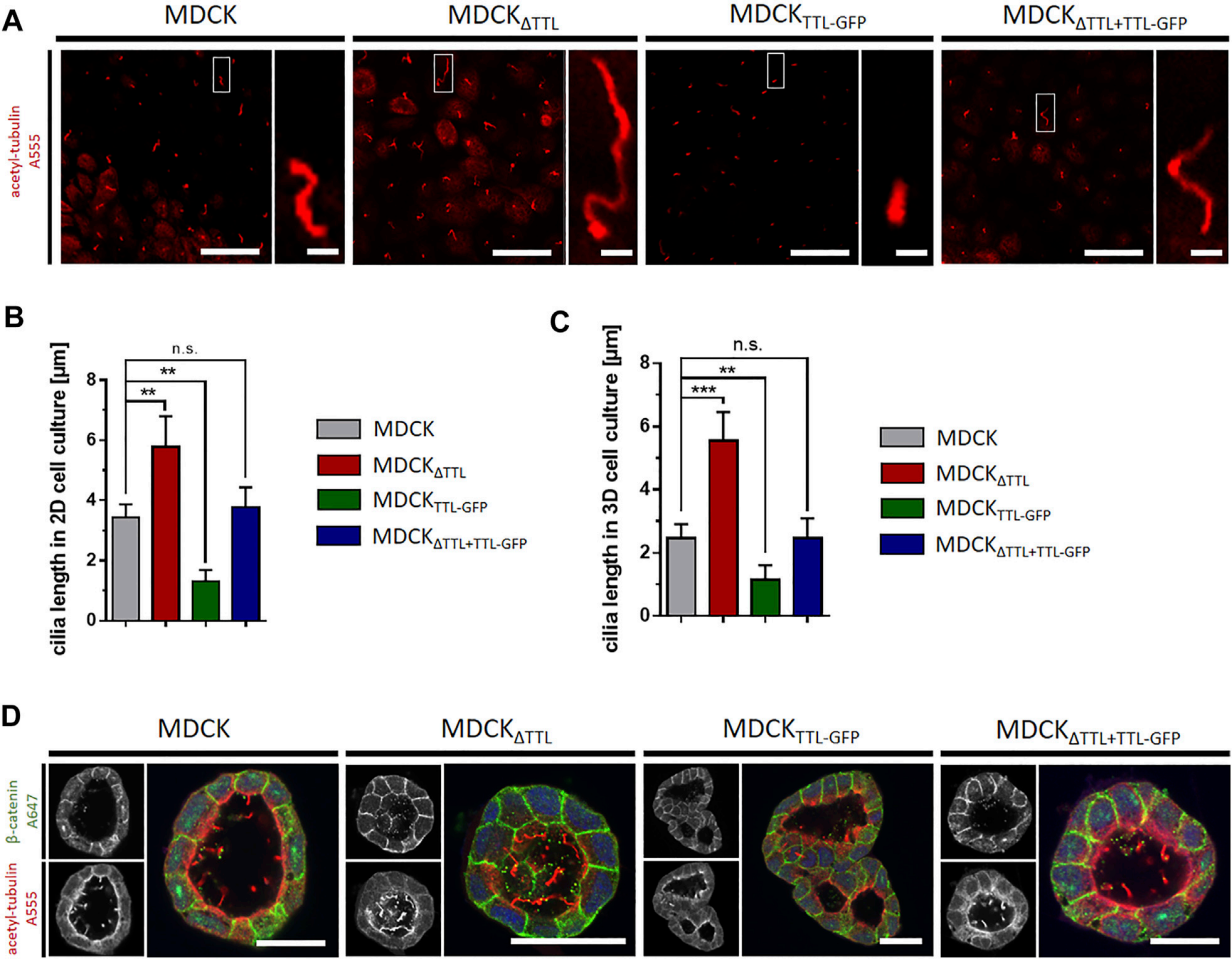
Primary cilia-length is affected by modulation of TTL-expression in 2D- and 3D-cell culture models. **(A)** The length of primary cilia immunostained with antibodies directed against acetylated tubulin (red) was measured from cells grown in monolayers on PET-filters. Scale bars: overviews, 20 μm; insets, 2 µm. **(B,C)** Quantification of primary cilia length in cells grown in monolayers on filters **(B)** and in cells grown in matrigel embedded cysts **(C)**. Mean ± s.d., *n* = 3. Statistical significance was tested using one-way ANOVA with Dunnet’s comparison (n.s., not significant; **p < 0.01, ***p < 0.001). **(D)** MDCK, MDCK_ΔTTL_, MDCK_TTL-GFP_ and MDCK_ΔTTL+TTL-GFP_ cells were cultured in Matrigel for 7 days. Cysts were stained with antibodies directed against β-catenin (green) and acetylated tubulin (red). Scale bars: 30 µm.

**FIGURE 8:**
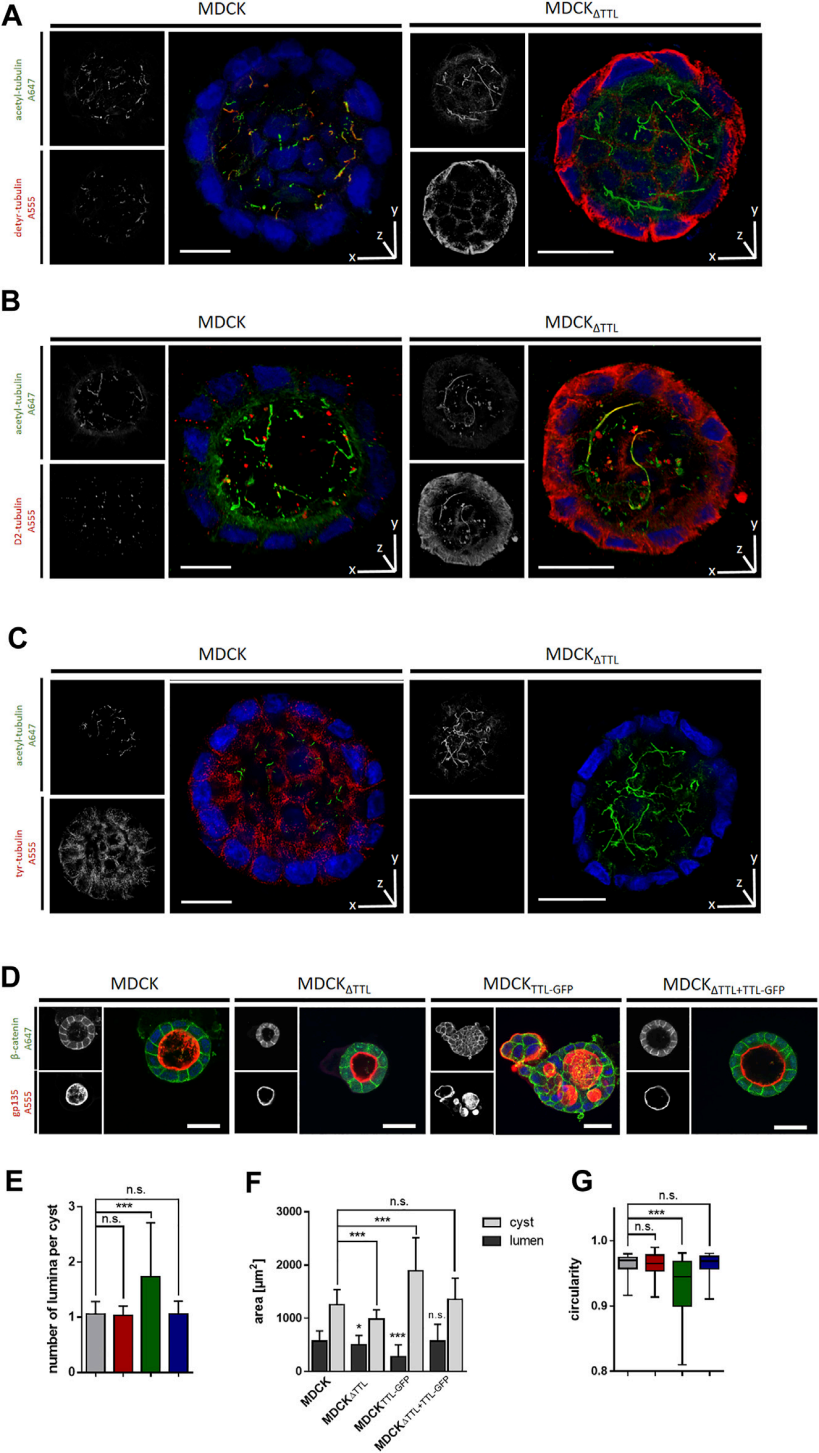
TTL-expression dependent tubulin PTMs in primary cilia. **(A–C)** 3D-rendered depiction of MDCK and MDCK_ΔTTL_ cells cultured in Matrigel for 7 days at 37°C and a humidified atmosphere of 5% CO_2_ in air. Cysts were stained with antibodies directed against acetylated tubulin (green) and detyr-tubulin (red) in **(A)**, D2-tubulin (red) in **(B)** and tyr-tubulin (red) in **(C)**. Distribution of tubulin PTMs were analyzed by confocal microscopy. Scale bars: 20 µm. **(D–G)** MDCK, MDCK_ΔTTL_, MDCK_TTL-GFP_ and MDCK_ΔTTL+TTL-GFP_ cells were cultured as described above. Polarity of cysts was analyzed by confocal microscopy. Cysts were stained with antibodies directed against lateral β-catenin (green) and apical gp135 (red). Scale bars: 30 µm. **(E–G)** Lumen numbers **(E)**, cyst and lumen areas **(F)** and the circularity of cysts **(G)** were quantified by using ImageJ analysis tools. Mean ± s.d., *n* = 3 independent experiments per cell line. A total of 7–10 cysts were analyzed per experiment and cell line. Mean ± s.d., *n* = 3. Statistical significance was tested using one-way ANOVA with Dunnet’s comparison (n.s., not significant; ***p < 0.001).

We then analyzed the mobility of EB1-plus-tips in polarized cells grown in a three-dimensional Matrigel matrix. Therefore, MDCK and MDCK_ΔTTL_ cells stably expressing EB1-GFP were generated (MDCK_EB1-GFP_, MDCK_ΔTTL/EB1-GFP_). Figure 6A depicts confocal views of circular grown cell cysts with apical surfaces facing the central lumen (Montesano et al., 1991). EB1-positive foci were detected in the cell cytoplasm. Most of these punctate structures moved from the apical to the basal cell pole, and their movement was recorded over time by live cell imaging (Figures 6B,C and Supplementary Movies S12, S13). Quantitative analysis revealed velocities of these EB1-GFP positive microtubule tips along the apicobasal axis, which are in similar ranges as recorded from cells migrating on a 2D-substratum (Figure 4). Again, microtubule plus-tips moved faster in MDCK_ΔTTL_ cells than in cells expressing TTL. This suggests that elevated detyr-tubulin concentrations facilitate plus end elongation of microtubules in not yet differentiated as well as in fully polarized epithelial cells. Moreover, some EB1-positive punctate structures moved towards the apical cell pole. We then focused on prominent locations of EB1-staining at the apical membrane domain. Here, EB1-GFP was found at the base of the primary cilium, which was labelled with antibodies directed against acetylated tubulin (Figure 6D). If we now used this antibody for immunofluorescence analysis of 2D cell monolayers (Figures 7A,B) or cysts formed by MDCK, MDCK_ΔTTL_, MDCK_TTL-GFP_ or MDCK_ΔTTL+TTL-GFP_ cells (Figures 7C,D), primary cilia of TTL-overexpressing MDCK_TTL-GFP_ cells were significantly stunted. On the other hand, primary cilia in MDCK_ΔTTL_ cells were about twice as long as in MDCK or MDCK_ΔTTL+TTL-GFP_ cells, which is an indicator for enhanced ciliogenesis. Co-staining between antibodies directed against acetylated and tyr-, detyr- or delta two tubulin (D2), an irreversible PTM of detyr-tubulin (Nieuwenhuis and Brummelkamp, 2019), revealed that among this selection primary cilia of MDCK cells were mainly composed of acetylated and detyr-tubulin (Figure 8A). Primary cilia of MDCK_ΔTTL_ cells, on the other hand, were enriched in acetylated and D2-tubulin (Figure 8B), which corresponds to a general increase of D2-tubulin in this cell line (Supplementary Figure S9) (Müller et al., 2021). Interestingly, detyr-tubulin, the precursor form of D2-tubulin was not detected in primary cilia of MDCK_ΔTTL_ cells. Also, tyr-tubulin was beyond the detection limit in primary cilia of MDCK_ΔTTL_ and MDCK cells (Figure 8C), which indicates first that acetylation and detyrosination but not re-tyrosination are preferred PTMs of α-tubulin in the axoneme. Second, there is a clear correlation between cilia length and removal of one or even two amino acids from the C-terminus of α-tubulin.

Ciliogenesis of primary cilia is known to play key roles in epithelial differentiation (Singla and Reiter, 2006). Consequently, we studied the polarized arrangement of MDCK cells in the 3D-cysts by staining of the apical membrane with anti-gp135 (podocalyxin) and of the lateral membrane with anti-β-catenin antibodies (Figures 8D–G). In contrast to the other cell lines, MDCK_TTL-GFP_ cells showed defects in cyst organization and developed multiple small lumens or tubes within the cysts (Figure 8E). These cysts were of elevated size and irregular shape with significantly lower levels of circularity (Figures 8F,G). Thus, lowering detyr-tubulin quantities in MDCK cells by TTL-overexpression results in the formation of extremely short primary cilia and perturbation of epithelial morphogenesis.

## Discussion

**FIGURE 9:**
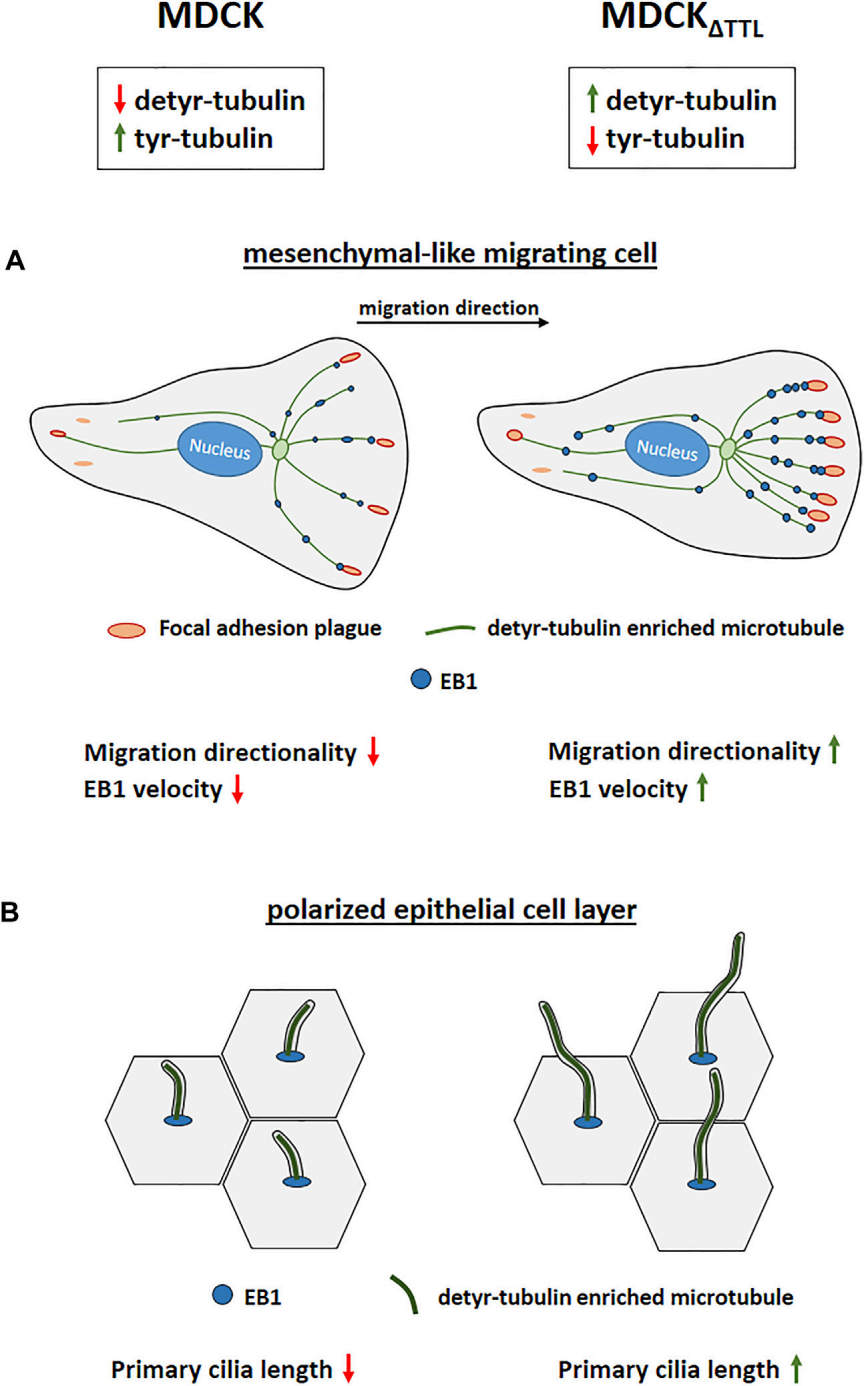
Modulation of TTL-expression alters directional cell migration and ciliogenesis. **(A)** Migrating MDCK cells devoid of TTL migrate with enhanced directionality. Elevated numbers of microtubules enriched in detyr-tubulin are decorated by EB1 and are in close proximity to focal adhesions placed in the direction of cell movement. **(B)** Polarized MDCK cells devoid of TTL exhibit exceptionally elongated primary cilia.

In this study we show, that epithelial cells devoid of TTL undergo gross changes in directed mobility as well as in epithelial morphogenesis (summarized in Figure 9). Enhanced directional organization of focal adhesions correlates with an increased mobility of the plus end binding protein EB1 in cells with microtubules enriched in detyrosinated tubulin. The EB1 distribution pattern thereby entirely shifted from prominent decoration of tyr-tubulin enriched microtubules in cells expressing TTL to detyr-tubulin enriched microtubules if TTL is absent. EB1 interaction with these microtubules may increase their stability as suggested by work on the EB1 homolog from fission yeast (Sandblad et al., 2006). It has also been shown that interaction of EB1 with the plus ends of detyrosinated microtubules facilitates and EB1 down-regulation or overexpression impairs cell migration (Wen et al., 2004; Scolz et al., 2012; Yang et al., 2017; Juanes et al., 2020), thus suggesting that EB1 capture stabilizes microtubule plus ends and provides anchoring sites for proteins involved in cell motility. Binding of EB1 to APC in the cortex of breast cancer cells controls APC-mediated actin assembly and, moreover, perturbation of normal EB1 levels, up or down, disrupts directional cell migration (Juanes et al., 2020). In addition, cell migration and focal adhesion dynamics are triggered by ubiquitylation of EB1 at focal adhesions (Courtheoux et al., 2016), which assigns EB1 the function of a master regulator in the organization of focal adhesions during cell migration. The observed increased velocities of EB1-positive comet-like plus-tips following TTL-knockout will elevate EB1 levels at focal adhesion plaques and may even link microtubules to these plaques. As a consequence, focal adhesion stability increases as determined by live-cell imaging and photoconversion experiments. Among the putative interaction partners of EB1 at these plaques is the integrin-ILK complex. This complex anchors microtubule plus ends to the basolateral surface of fully polarized epithelial cells to EB1 (Akhtar and Streuli, 2013). Our study suggests that the integrin-ILK complex fulfills similar functions in migrating epithelial cells that have not yet undergone apicobasal polarization. ILK interacts with the cytoplasmic domains of β1 and β3 integrin subunits (Hannigan et al., 1996) as well as with tubulin and various mitotic spindle associated proteins (Fielding et al., 2008). Association between EB1, detyr-tubulin and ILK exposes this complex as a prime candidate for mediators between posttranslationally modified microtubules and focal adhesion plaques.

Alterations following TTL-knockdown are not restricted to epithelial migration. The development of MDCK cell cysts in a 3D-gel matrix is also affected. Based on observations showing that EB1 is required for epithelial remodeling in 3D (Gierke and Wittmann, 2012), we located this polypeptide on microtubules growing in the direction of the apical or basal cell pole and at the base of the primary cilium, which is in agreement with observations in fibroblasts and RPE cells (Schrøder et al., 2007; Schrøder et al., 2011). Microtubules anchoring at the base of the primary cilium may thus serve as tracks for vesicles carrying ciliary components that release their cargo at the basal body. In view of the described function of EB1 in the assembly of primary cilia it is obvious to conclude that the increased velocities of EB1-plus-tips in polarized epithelial cells devoid of TTL as described in this study will also affect the organization of the primary cilium. Local elevation of EB1 at basal bodies could represent an increase in microtubule anchoring or nucleation, as EB1 is involved in both (Yan et al., 2006). Indeed, we found significant elongation of the length of these cilia in TTL-knockout MDCK cells grown in 2D monolayers or in 3D cysts. The cilia were enriched in D2 tubulin, a tubulin variant accumulated in TTL^−/−^ mice (Erck et al., 2005). It is unlikely that this modification per se stabilizes microtubules for cilia elongation since the PTM-dependent recruitment of regulator proteins is central in the steering of microtubule dynamics (Chen et al., 2021). Accordingly, various cellular processes, including actin dynamics and endocytic trafficking are involved in the complex regulation of cilia assembly/disassembly (Dawe et al., 2007; Kim et al., 2010; Pitaval et al., 2010). Further evidence suggests that the regulation of cilia length encompasses components of the cellular machinery for autophagy (Pampliega et al., 2013; Tang et al., 2013). Findings describing that microtubule stabilization drives the initiation of ciliogenesis (Pitaval et al., 2017) and that the loss of ciliogenesis genes increases cytosolic PTMs of α-tubulin (Berbari et al., 2013) reveal a scenario in which ciliogenesis and tubulin-modification are ultimately linked to each other. As a consequence, alterations in the tubulin code would influence some of the phenotypes observed in ciliopathies. Epithelial morphogenesis relies on a functional primary cilium. The disruption of primary cilia or mutations in cilia-associated proteins results in renal epithelial proliferation and cyst growth in animal models and human genetic diseases that are classified as ciliopathies (Hildebrandt et al., 2011), for instance characteristic of the development of the autosomal recessive polycystic kidney disease (APKD) [(Singla and Reiter, 2006); (Kolb and Nauli, 2008)]. Here, we show that, upon TTL-overexpression, stunted primary cilia result in abnormal cystogenesis in MDCK cells. In APKD mouse models stunted (Murcia et al., 2000) as well as elongated cilia (Husson et al., 2016) have been observed on tubular epithelial cells. Stunted primary cilia have defects in intracellular signalling of hedgehog, TGF-β and Wnt pathways (Anvarian et al., 2019). It thus seems plausible that the dramatic shortening of primary cilia observed in TTL-overexpressing MDCK cells alters epithelial differentiation and disrupts the coordinated sequence of events in 3D cyst formation.

Finally, the primary cilium also plays significant roles in cell migration so that defects in their assembly/disassembly affect the movement and placement of cells (Veland et al., 2014). Thus, we can not exclude putative roles of the impairments of ciliogenesis on the observed changes in cell migration. This will have to be clarified in future studies.

## Data Availability

The original contributions presented in the study are included in the article/Supplementary Material, further inquiries can be directed to the corresponding author.
