# Supplementary material for: Manipulation of the Tubulin Code Alters Directional Cell Migration and Ciliogenesis
Source: Front Cell Dev Biol. 2022 Jul 12;10:901999. doi: 10.3389/fcell.2022.901999 (PMC9315229; doi:10.3389/fcell.2022.901999)
Supplement: Supplementary file 3 [file DataSheet1.pdf]

Supplementary Material

Figure S1

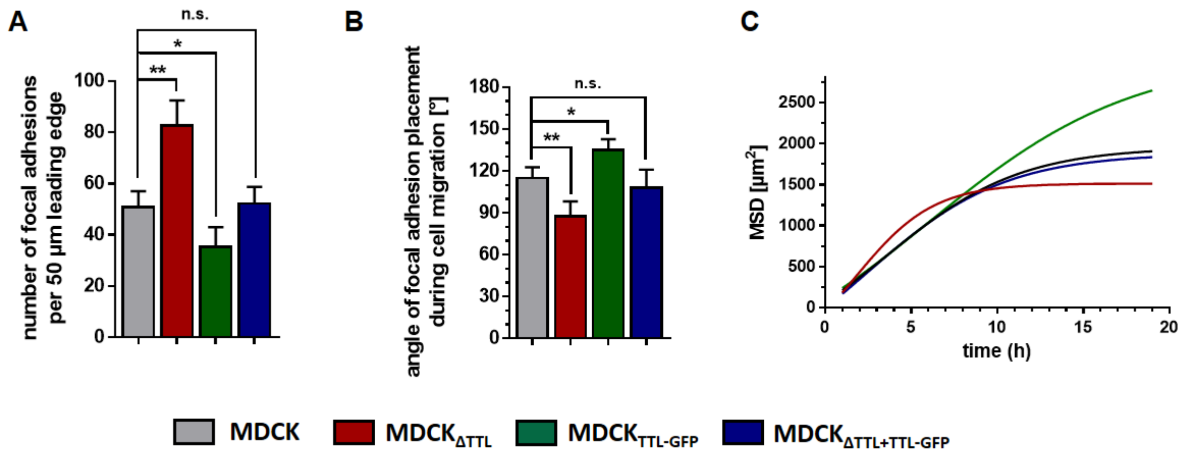

Figure S1: Quantification of focal adhesions replacement during cell migration.

(A) MDCK, MDCK$_{\Delta TTL}$, MDCK$_{TTL\text{-}GFP}$ and MDCK$_{\Delta TTL+TTL\text{-}GFP}$ were stained after 6 h of migration as described in figure 1. Number of focal adhesions per 50 µm leading edge were counted and quantified for each cell line. Mean±s.d., n=3 independent experiments per cell line. Statistical significance was tested using one-way ANOVA with Dunnet's comparison (n.s., not significant; *$P<0.05$; **$P<0.01$). (B) Quantification of focal adhesion placement angle during cell migration in each cell line. Angles were measured by ImageJ. A total of 10-15 cells were analyzed per experiment. Mean±s.d., n=3 independent experiments per cell line. Statistical significance was tested using one-way ANOVA with Dunnet's comparison (n.s., not significant; *$P<0.05$; **$P<0.01$). (C) For MSD plots spline curves from interpolated ImageJ tracking analysis of 4 cells per experiment were generated. n=6 independent experiments per cell line.

Figure S2

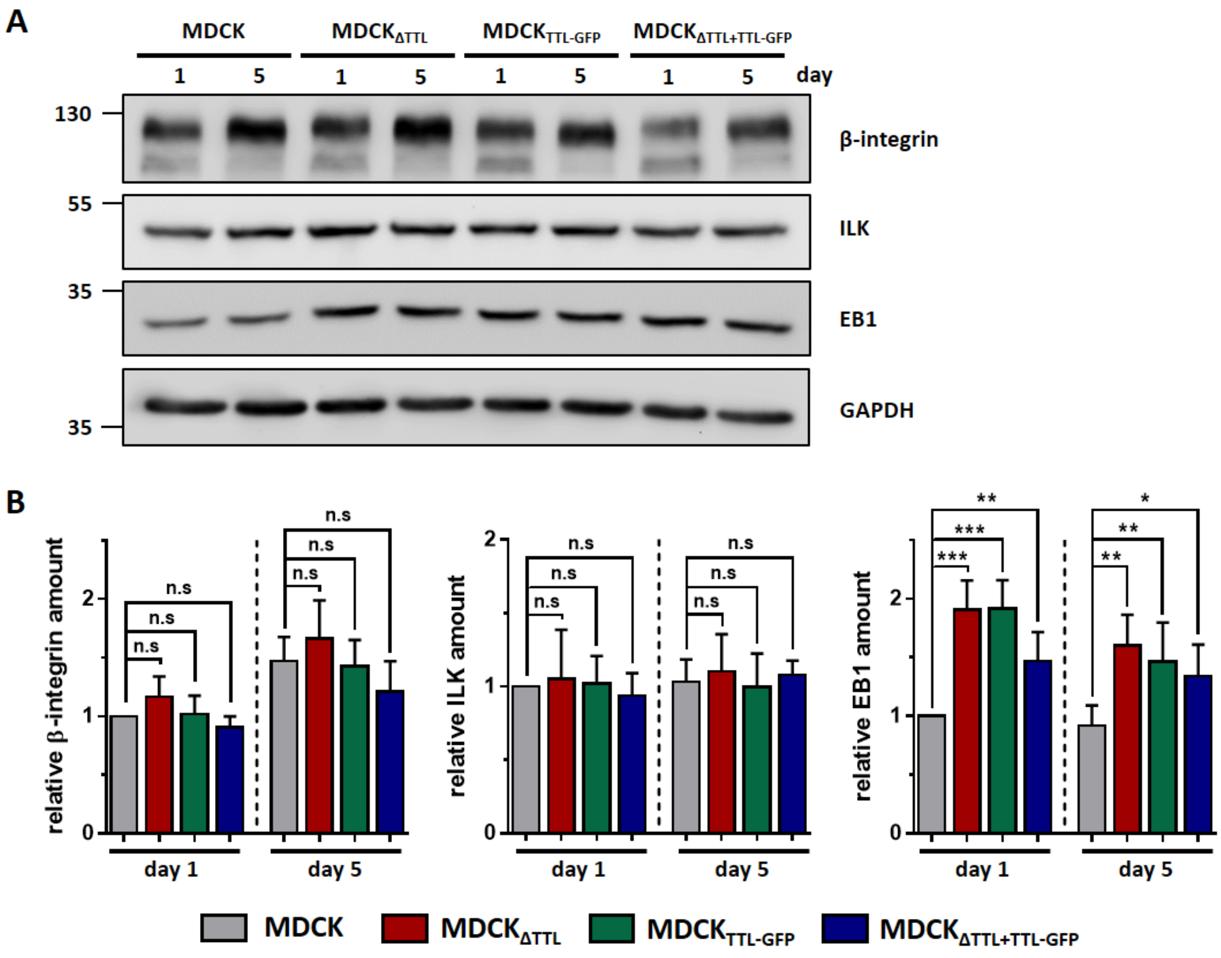

Figure S2: Expression profiles of β1-integrin, ILK and EB1 in MDCK, MDCK$_{\Delta TTL}$, MDCK$_{TTL\text{-}GFP}$ and MDCK$_{\Delta TTL+TTL\text{-}GFP}$ cells.

(A, B) Lysates of subconfluent and confluent MDCK, MDCK$_{\Delta TTL}$, MDCK$_{TTL\text{-}GFP}$ and MDCK$_{\Delta TTL+TTL\text{-}GFP}$ cells were analyzed by immunoblot with antibodies directed against β1-integrin (CD29), ILK and EB1. Equal amounts (20 µg) of lysates were loaded. GAPDH served as a loading control. Representative results. (B) Relative quantities were normalized to GAPDH levels in cell lysates. Mean±s.d., n=3. Statistical significance was tested using one-way ANOVA with Dunnet's comparison (n.s., not significant; *P<0.05; **P<0.01; ***P<0.001).

Figure S3

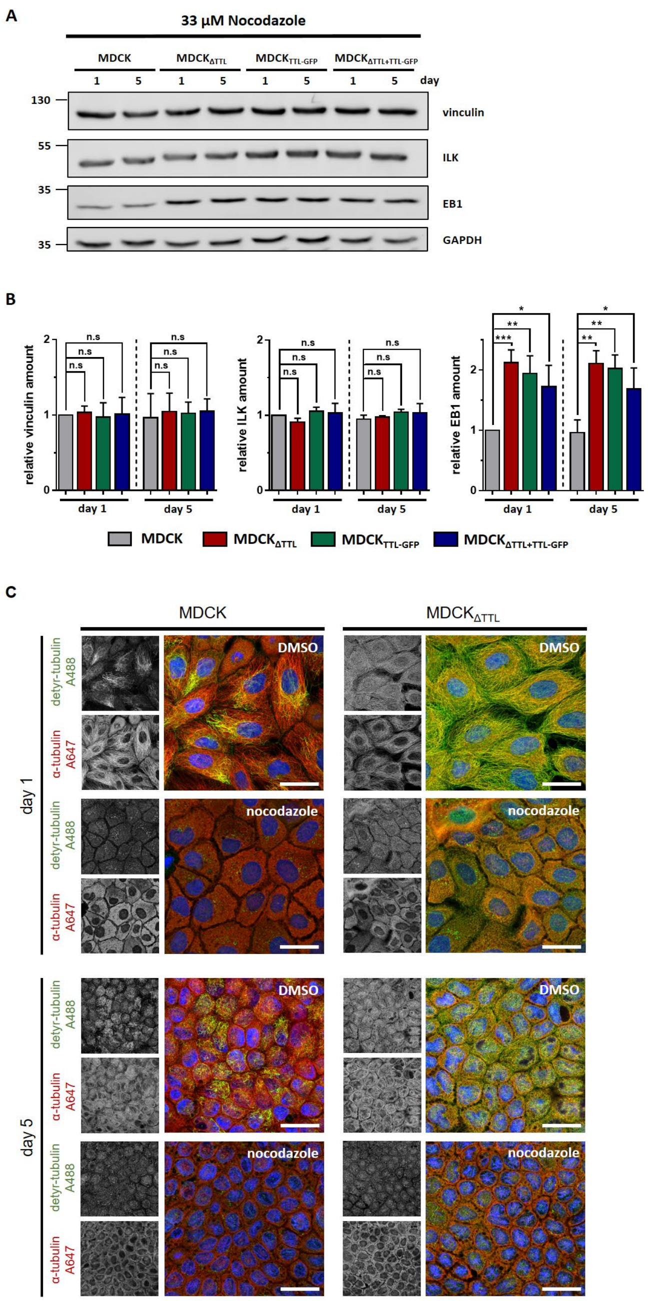

Figure S3: Effect of nocodazole treatment on the expression patterns of vinculin, ILK and EB1.

(A, B) Lysates of subconfluent and confluent MDCK, MDCK$_{\Delta TTL}$, MDCK$_{TTL\text{-}GFP}$ and MDCK$_{\Delta TTL+TTL\text{-}GFP}$ cells, treated with 33 µM nocodazole for 1 h on ice and 1 h at 37°C, were analyzed by immunoblot with antibodies directed against vinculin, ILK and EB1. Equal amounts of lysates were loaded. GAPDH served as a loading control. Representative results. (B) Relative quantities were normalized to GAPDH in cell lysates. Mean±s.d., n=3. Statistical significance was tested using one-way ANOVA with Dunnet's comparison (n.s., not significant; *P<0.05; **P<0.01; ***P<0.001). (C) Successful disruption of microtubules following nocodazole-treatment was analyzed in non-polarized and polarized MDCK and MDCK$_{\Delta TTL}$ cells. The cells were fixed and stained for α-tubulin (red) and detyr-tubulin (green). Scale bars: 30 µm.

Figure S4

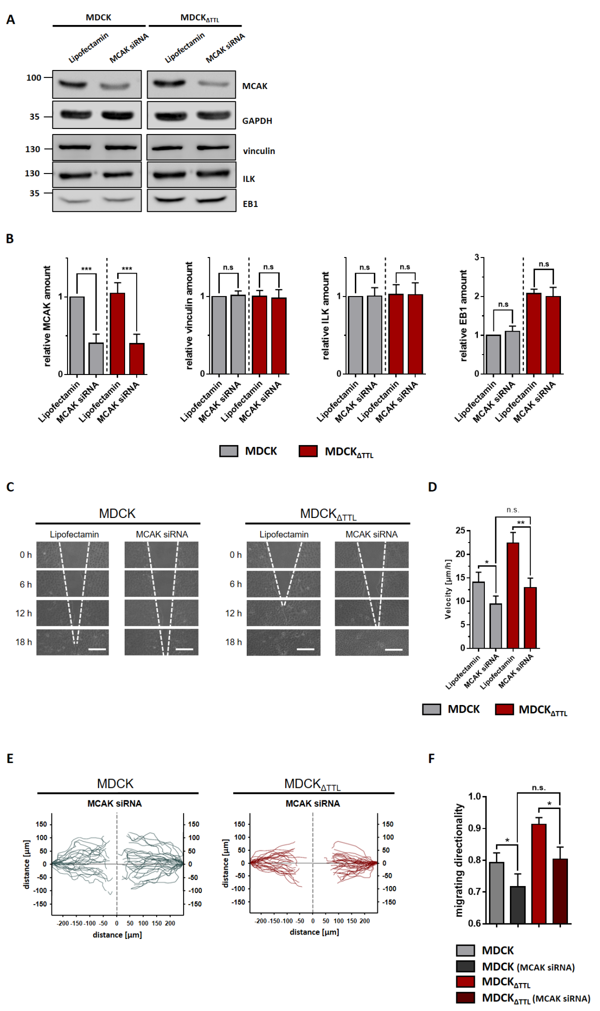

Figure S4: MCAK-knockdown leads to delayed cell migration after TTL-modulation.

(A) MDCK and MDCK$_{\Delta TTL}$ cells were transfected with MCAK-specific siRNA. Lysates of control cells (lipofectamin treated) and MCAK-depleted cells were analyzed by immunoblot with antibodies directed against MCAK, vinculin, ILK and EB1. GAPDH served as a loading control. n=3 independent experiments. Representative result. (B) Relative quantities were normalized to GAPDH levels in cell lysates. Mean±s.d., n=3. Statistical significance was tested using one-way ANOVA with Dunnet's comparison (n.s., not significant; ***P<0.001). (C) Confluent MDCK and MDCK$_{\Delta TTL}$ cell-monolayers of MCAK-depleted and control cells (lipofectamin treated) were scratch wounded to analyze wound closure. Cells were recorded at 0, 6, 12 and 18 h post-scratching. White dotted lines indicate the wound borders progress. Scale bars: 100 µm. (D) Mean cell migration velocity was calculated. Mean±s.d., n=3. Statistical significance was tested using one-way ANOVA with Dunnet's comparison (n.s., not significant; *P<0.05; **P<0.01). (E) Single cell migration was recorded and visualized by Tracking Tool^{TM} PRO (Gradientech). One line corresponds to one single cell track of each cell line. (F) Cell migration directionality was calculated by Tracking Tool^{TM} PRO. Mean±s.d., n=3 independent experiments per cell line were performed. Statistical significance was tested using one-way ANOVA with Dunnet's comparison (n.s., not significant; *P<0.05).

Figure S5

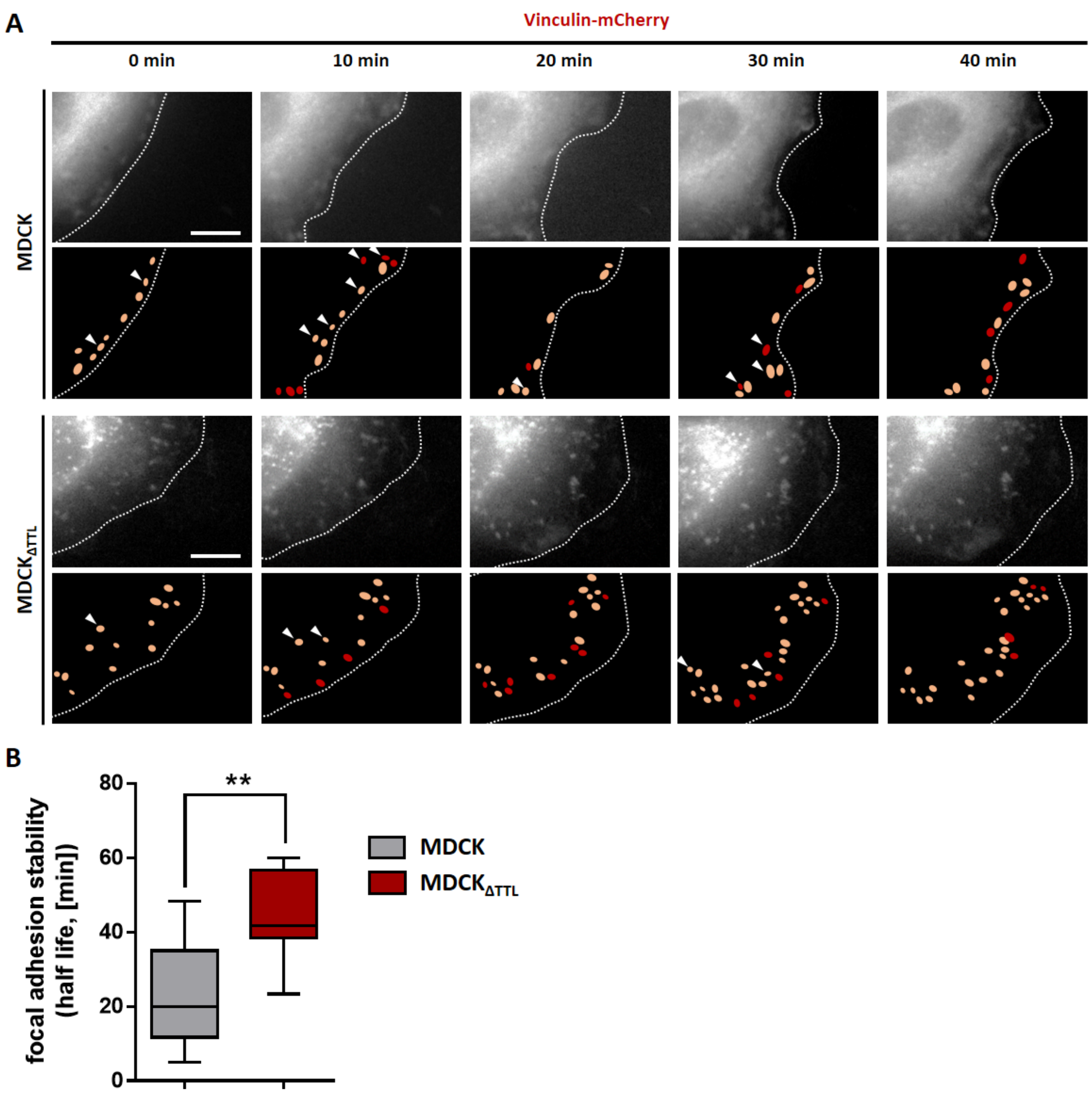

Figure S5: Stability of vinculin-mCherry labelled FAs in MDCK cells.

(A) Migrating MDCK and MDCK$_{\Delta TTL}$ cells transiently transfected with vinculin-mCherry incubated on glass bottom WillCo dishes under a humidified atmosphere of 5% CO_2 in air. Assembly and disassembly of focal adhesions were analyzed by fluorescence microscopy at 37 °C. Vinculin-mCherry fluorescence after varying time intervals is indicated in the upper rows and identified FAs are depicted in the lower rows. Newly formed FAs per time interval are shown in red, FAs fading away in one time interval are indicated by arrowheads, remaining FAs are labelled in orange. White dotted lines indicate the migration progress. Scale bars, 10 µm. (B) Quantification of FAs half-lives in MDCK and MDCK$_{\Delta TTL}$ cells. Mean±s.d., n=3. Statistical significance was tested with Student`s unpaired t-test (**$P<0.01$).

Figure S6

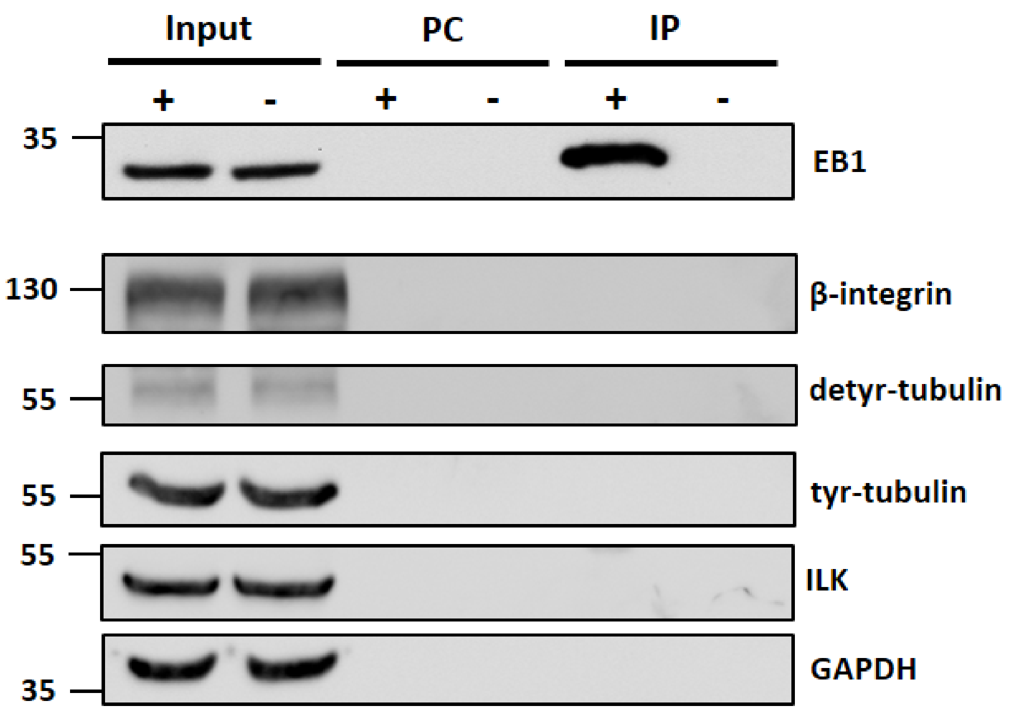

Figure S6: TTL-overexpression reduces pulldown of EB1 with tubulin and ILK.

$MDCK_{TTL\text{-}GFP}$ cell lysates were incubated with anti-EB1 antibodies followed by precipitation with agarose beads. Precipitates were analyzed by immunoblot using antibodies directed against β1-integrin, detyr-tubulin, tyr-tubulin, ILK, GAPDH and EB1. Representative results, n=3 independent experiments. PC, Pre-clearing using agarose beads; IP+, Immunoprecipitation using EB1 antibodies and agarose beads; IP-, Immunoprecipitation using unspecific IgG antibodies and agarose beads.

Figure S7

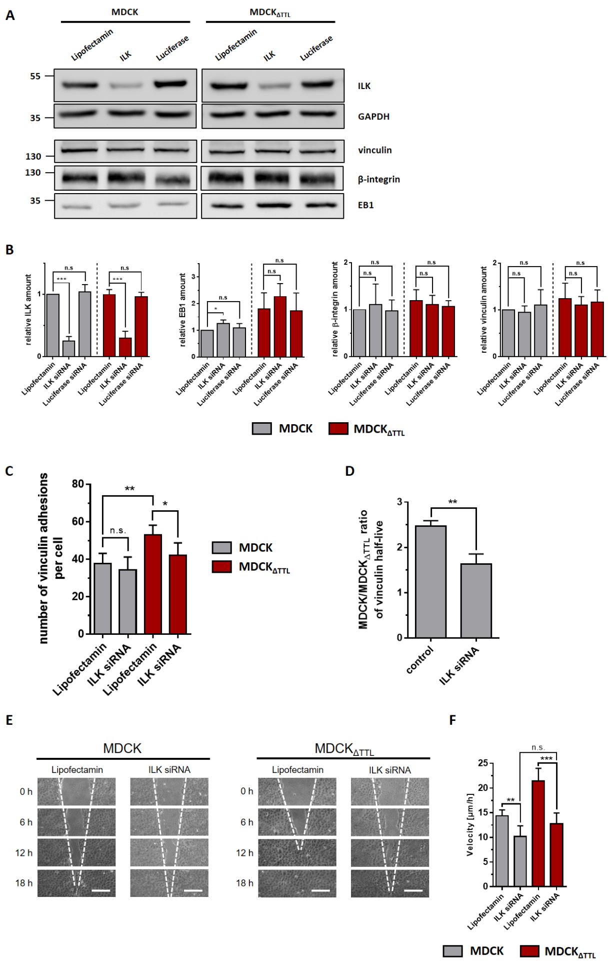

Figure S7: ILK-knockdown delays cell migration and reduces focal adhesion quantities after TTL-modulation.

(A) MDCK and MDCK$_{\Delta TTL}$ cells were transfected with ILK-specific siRNA overnight. Lysates of control cells (lipofectamin treated), ILK-depleted cells and luciferase-siRNA treated cells were analyzed by immunoblot with antibodies directed against integrin linked kinase (ILK), vinculin, β1-integrin (CD29) and EB1. Equal amounts (20 µg) of lysates were loaded. GAPDH served as a loading control. n = 3 independent experiments. Representative result. (B) Relative quantities were normalized to GAPDH levels in cell lysates. Mean±s.d., n=3. Statistical significance was tested using one-way ANOVA with Dunnet's comparison (n.s., not significant; *P<0.05; ***P<0.001). (C) Quantification of vinculin-positive focal adhesions per cell. Comparison of cells after knockdown of ILK and control cells (lipofectamin treated). Mean±s.d., 8 – 10 cells per experiment, n=3 independent experiments. Statistical significance was tested using one-way ANOVA with Dunnet's comparison (n.s., not significant; *P<0.05; **P<0.01). (D) Quantification of MDCK/MDCK$_{\Delta TTL}$ vinculin half-life ratio. Mean±s.d., n=3. Statistical significance was tested with Student`s unpaired t-test (**P<0.01). (E) Confluent MDCK and MDCK$_{\Delta TTL}$ cell-monolayers of ILK-depleted and control cells (lipofectamin treated) were scratch wounded to analyze migration velocity. Cells were recorded at 0, 6, 12 and 18 h post-scratching. White dotted lines indicate the wound borders progress. Scale bars: 100 µm. (F) Mean cell migration velocity was calculated. Mean±s.d., n=5. Statistical significance was tested using one-way ANOVA with Dunnet's comparison (n.s., not significant; **P<0.01; ***P<0.001).

Figure S8

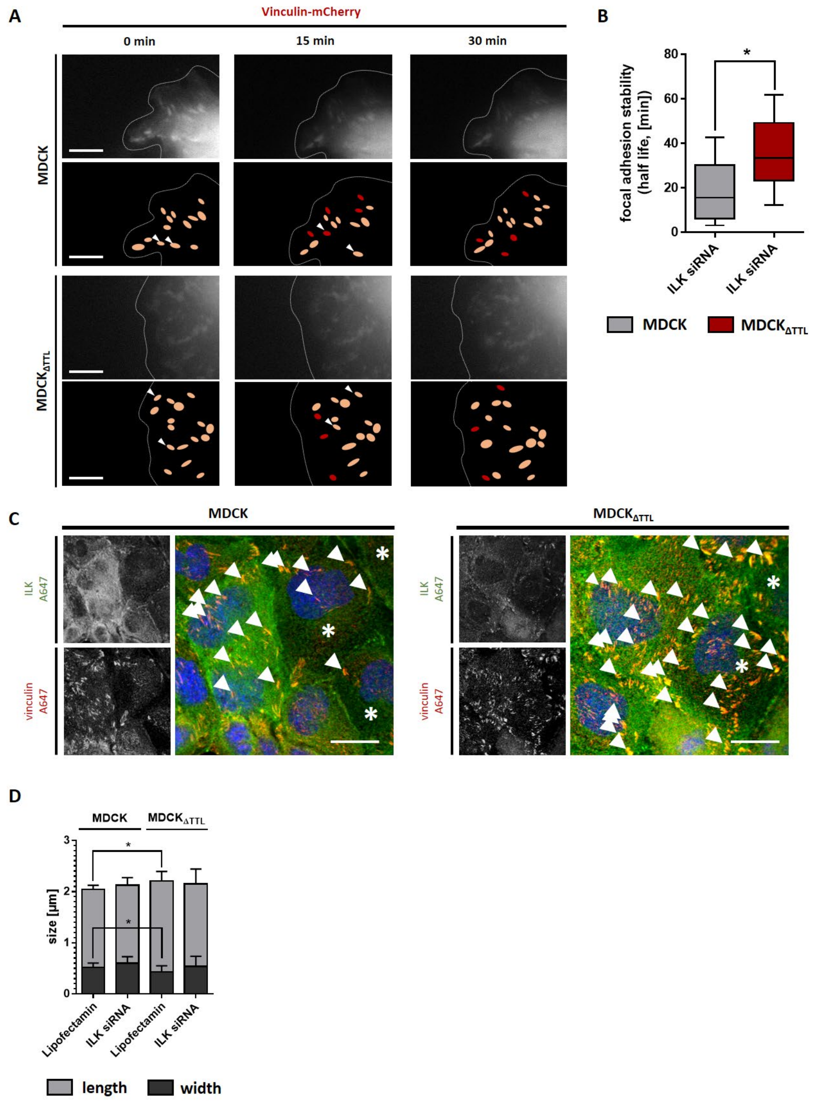

Figure S8: ILK-knockout changes dynamic and size of focal adhesions.

(A) Migrating ILK-depleted MDCK and MDCK$_{\Delta TTL}$ cells transiently transfected with vinculin-mCherry were analyzed by fluorescence microscopy at 37 °C. Vinculin-mCherry fluorescence after varying time intervals is indicated in the upper rows and and identified FAs are depicted in the lower rows. Newly formed FAs per time interval are shown in red, FAs fading away in one time interval are indicated by arrowheads, remaining FAs are labelled in orange. White dotted lines indicate the migration progress. Scale bars, 8 µm. (B) Quantification of FAs half-lives in MDCK and MDCK$_{\Delta TTL}$ cells. Mean±s.d., n=3. Statistical significance was tested with Student`s unpaired t-test (*P<0.05). (C) ILK-depleted MDCK and MDCK$_{\Delta TTL}$ cells were immunostained for ILK (green) and vinculin (red) Scale bars: 10 µm. Arrowheads indicate exemplary vinculin spots. Asterisks indicate cells with down knocked ILK. (D) Quantification of FAs (vinculin) length (light grey) and width (dark grey) in MDCK and MDCK$_{\Delta TTL}$ cells following ILK-knockdown by siRNA. Mean±s.d., n=3 independent experiments per cell line. Statistical significance was tested using one-way ANOVA with Dunnet's comparison (*P<0.05).

Figure S9

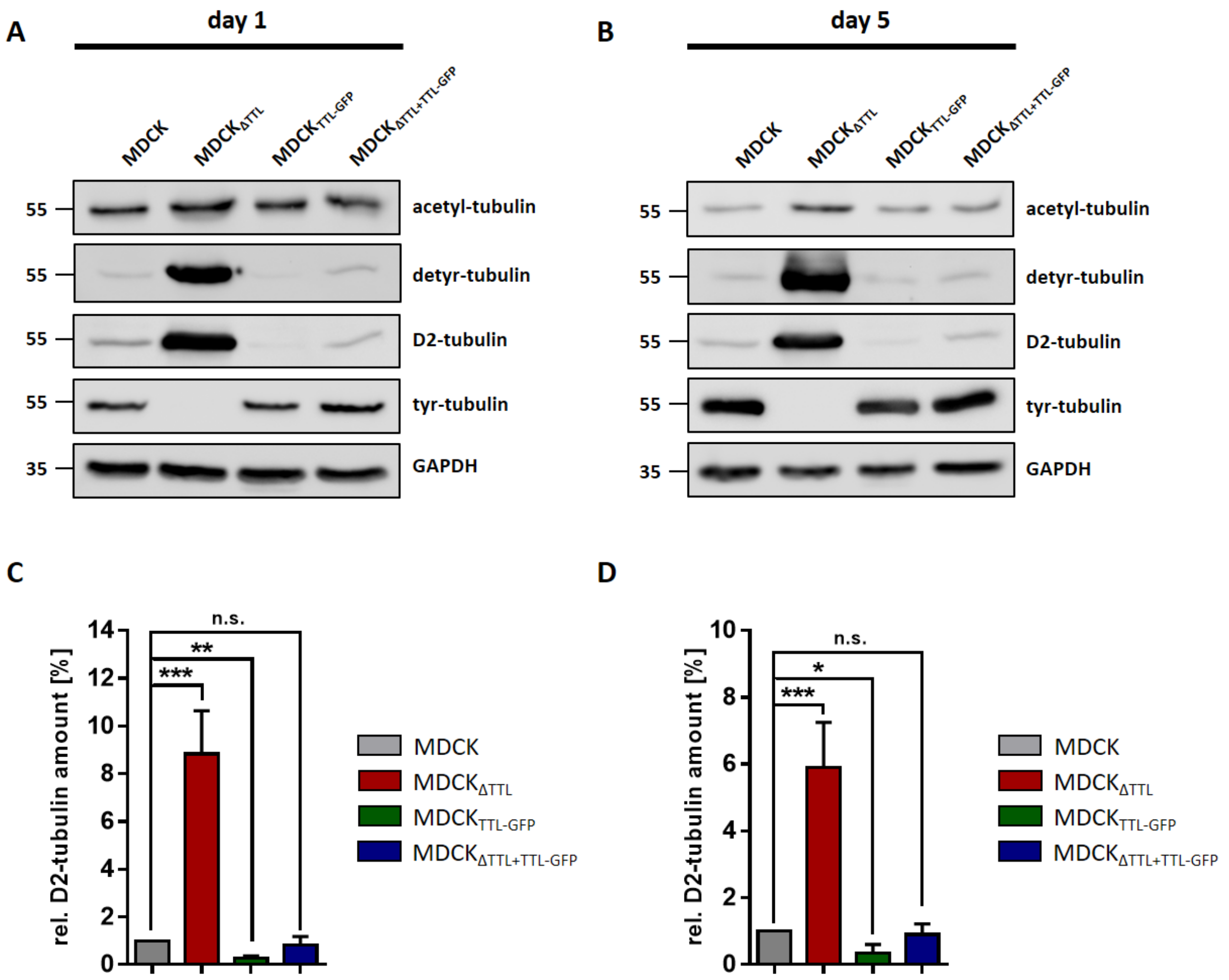

Figure S9: TTL knockout leads to increased amount of D2-tubulin.

(A,B) Cellular levels of acetyl-tubulin, detyr-tubulin, D2-tubulin and tyr-tubulin were assessed by Western blot analysis of cell lysates from non-polarized (A) and polarized (B) MDCK, $MDCK_{\Delta TTL}$, $MDCK_{TTL\text{-}GFP}$, and $MDCK_{\Delta TTL+TTL\text{-}GFP}$ cells. Protein concentrations of the lysates were determined and equal amounts were loaded on each lane of the SDS-PAGE. Representative results. (C,D) Quantification of relative D2-tubulin expression in each cell line as compared to MDCK cells for non-polarized (C) and polarized (D) cells. Relative polypeptide expressions were normalized to GAPDH levels. Quantities from MDCK cells were set as 1. Mean±s.d., n=3. Statistical significance was tested using one-way ANOVA with Dunnet's comparison (n.s., not significant; *P<0.05; **P<0.01; ***P<0.001).

Movie S10: EB1-dynamics in MDCK.

Subconfluent living MDCK cells transiently transfected with EB1-GFP incubated on glass bottom WillCo dishes under a humidified atmosphere of 5% CO_2 in air. Dynamic of EB1-GFP (grey) was analyzed by fluorescence microscopy at 37 °C. Time-lapse imaging was recorded for 60 sec. Scale bar: 5 µm.

Movie S11: EB1-dynamics in $MDCK_{\Delta TTL}$.

Subconfluent living $MDCK_{\Delta TTL}$ cells transiently transfected with EB1-GFP incubated on glass bottom WillCo dishes under a humidified atmosphere of 5% CO_2 in air. Dynamic of EB1-GFP (grey) was analyzed by fluorescence microscopy at 37 °C. Time-lapse imaging was recorded for 60 sec. Scale bar: 5 µm.

Movie S12: Basal and apical dynamics of EB1 in MDCK cysts.

Living MDCK cells stably transfected with EB1-GFP were cultured in Matrigel for 7 days. Dynamics of EB1-GFP (grey) was analyzed by fluorescence microscopy at 37 °C and a humidified atmosphere of 5% CO_2 in air. Time-lapse imaging was recorded for 51 sec. Each arrow indicates EB1-GFP comet-like movement orientated to the apical membrane. Scale bar: 20 µm.

Movie S13: Basal and apical dynamics of EB1 in $MDCK_{\Delta TTL}$ cysts.

Living $MDCK_{\Delta TTL}$ cells stably transfected with EB1-GFP were cultured in Matrigel for 7 days. Dynamics of EB1-GFP (grey) was analyzed by fluorescence microscopy at 37 °C and a humidified atmosphere of 5% CO_2 in air. Time-lapse imaging was recorded for 63 sec. Arrow indicates EB1-GFP comet-like movement orientated to the apical membrane. Scale bar: 20 µm.

Table 1

	MDCK	MDCK$_{\Delta TTL}$	MDCK$_{TTL\text{-}GFP}$	MDCK$_{\Delta TTL+TTL\text{-}GFP}$
n	velocity	velocity	velocity	velocity
1	12,0	24,0	9,0	13,9
2	12,0	22,2	8,5	13,6
3	16,0	20,4	12,3	16,5
4	13,4	20,7	8,4	12,8
5	13,4	23,9	7,3	16,5
6	14,3	24,4	11,0	12,7
**mean**	**13,52**	**22,60**	**9,42**	**14,33**
**SD**	**± 1,38**	**± 1,61**	**± 1,70**	**± 1,59**

Table 1: Migration velocity in wound healing assay.

Migration velocity [µm/h] of MDCK, MDCK$_{\Delta TTL}$, MDCK$_{TTL\text{-}GFP}$ and MDCK$_{\Delta TTL+TTL\text{-}GFP}$. Cells were incubated on a glass bottom Wilco Dish to form a confluent monolayer. Scratches were added by sterile micropipette tip. Cell migration experiments were recorded on a PAULA microscope (Personal Automated Lab Assistant, Leica Microsystems). Scratch closure rates were recorded to deduce migration velocities by monolayer edge velocimetry (ImageJ analysis tools). n=6. SD=standard deviation.
